# Assessing the impact of TET2 and TET3 deletion in TCRalpha and TCRbeta repertoire in murine CD4 T cells in physiological and pathophysiological conditions

**DOI:** 10.3389/fimmu.2025.1638500

**Published:** 2025-08-20

**Authors:** Tarmo Äijö, Marianthi Gioulbasani, Jair Ernesto Valenzuela, Ageliki Tsagaratou

**Affiliations:** ^1^ Lineberger Comprehensive Cancer Center, University of North Carolina at Chapel Hill, Chapel Hill, NC, United States; ^2^ School of Biology, Aristotle University of Thessaloniki, Thessaloniki, Greece; ^3^ Lampe Joint Department of Biomedical Engineering, University of North Carolina at Chapel Hill, Chapel Hill and North Carolina State University, Raleigh, NC, United States; ^4^ Department of Genetics, University of North Carolina at Chapel Hill, Chapel Hill, NC, United States; ^5^ Department of Microbiology and Immunology, University of North Carolina at Chapel Hill, Chapel Hill, NC, United States

**Keywords:** TET proteins, TCR sequencing, TCR diversity, TCR clonality, TCR oligoclonal expansion

## Abstract

Ten Eleven Translocation (TET) proteins can oxidize 5-methylcytosine to generate in sequential steps oxidized forms of cytosine: 5-hydroxymethylcytosine, 5-formylcytosine and 5-carboxylcytosine. Through their catalytic activity TET proteins promote active DNA demethylation. There are three TET proteins: TET1, TET2 and TET3. In T cells, TET2 and TET3 are more highly expressed. In the past years we have extensively analyzed the impact of TET proteins and 5-hydroxymethylcytosine in T cell development. In this report, we focus on the impact of TET proteins in the TCR alpha (α) and beta (β) repertoires in thymic CD4 single positive cells and upon migration in the periphery. Our data reveal that both wild type and *Tet2/3* DKO CD4 cells in the thymus and the spleen are polyclonal. Then, we focus on *Tet2/3* DKO CD4 cells that are serially transplanted in recipient mice. Our TCR sequencing data reveals that expanded *Tet2/3* DKO CD4 cells are less diverse and oligoclonal. Overall, this report serves as a resource of TCRα and TCRβ repertoire in both wild type and *Tet2/3* DKO murine conventional CD4 T cells and provides insights on how expanded *Tet2/3* DKO CD4 cells opt for specific TCRα and β repertoires.

## Introduction

TET proteins are iron(II)/α-ketoglutarate (Fe(II)/α-KG)-dependent dioxygenases that catalyze the oxidation of 5-methylcytosine (5mC) to 5-hydroxymethylcytosine (5hmC) (1). There are three TET proteins; TET1, TET2 and TET3. *TET1* was identified as a fusion partner of *MLL* in a case of pediatric acute myeloid leukemia (AML) bearing the t(10;11)(q22;q23) translocation (2, 3). All three TET proteins share a common, evolutionary conserved carboxyl-terminal catalytic domain that consists of a double-stranded β-helix domain and a cysteine-rich domain (4). Via their catalytic domain TET proteins oxidize 5-methylcytosine (5mC) to 5-hydroxymethylcytosine (5hmC) (1) and other oxidized cytosines (oxi-mCs), namely 5-formylcytosine (5fC) and 5-carboxylcytosine (5caC) (5). Loss of 5hmC correlates with myelodysplastic syndromes (6). *Tet2* is one of the most frequently mutated genes in hematological cancers (7–9), such as angioimmunoblastic T cell lymphoma (AITL) (10–12) and peripheral T cell lymphoma, non-otherwise specified (PTCL-NOS) (13). TET2 mutations occur early in hematopoietic stem cells (HSCs) and *Tet2* mutant HSCs proliferate faster compared to wild type HSCs, however *Tet2* mutant HSCs are not fully transformed (7, 9, 13). Instead, secondary mutations are required to result in cancer emergence (9, 14). Further research using mice, demonstrated that murine *Tet2* deficient HSCs proliferate faster compared to wild type HSCs *in vivo* and *in vitro* in serial plating assays (15, 16). This enhanced proliferative capacity of *Tet2* HSCs was dependent on the enzymatic activity of TET2 (17, 18). Interestingly, targeting specifically TET2 in murine models is not sufficient to result in T cell expansion, presumably due to redundant functions with other TET proteins (19, 20).

Our work the past years has demonstrated a critical role for TET proteins and 5hmC in T cell development and differentiation (21, 22). Specifically, we (21) and others (23) have demonstrated that 5hmC marks active enhancers and decorates the gene body of highly expressed genes during thymic development in conventional (21, 24) and unconventional T cells (22, 24), such as invariant natural killer T (iNKT) cells, as well as in peripheral naïve and helper T cells (21, 23), and in regulatory T cells (Tregs) (25, 26). Importantly, it has been demonstrated that 5hmC is critical for recruiting DNA repair proteins at loci of DNA damage and in particular at double strand breaks (DSBs) (27). It has also been shown that oxi-mCs are stable epigenetic marks (28, 29) that are preferentially recognized by DNA repair proteins (30–32). Along these lines, immune cells that lack TET proteins such as hematopoietic stem cells, B cells, myeloid cells, iNKT cells and conventional T cells demonstrate genomic instability, defects in DNA repair and increase in DSBs (20, 22, 32–35).

We have previously systematically studied the thymic development of TET-deficient conventional and unconventional T cells (9, 36–39). *Tet2/3* DKO iNKT cells in the thymus are significantly increased and they are characterized by skewing towards the NKT17 lineage (22, 39). This phenotype is regulated by TET2 catalytic activity (24). We have demonstrated that *Tet2/3* DKO CD4 SP cells that develop in the thymus exhibit reduced levels of ThPOK, due to increased methylation of the proximal enhancer and the intragenic site A (24). Site A is bound by GATA3 in wild type CD4 SP cells when site A is unmethylated and can potentially function as a regulatory element to impact ThPOK expression (40). TET proteins are implicated in Treg stability (25, 41–44) and Treg function (26, 45, 46). In addition, we have demonstrated that *Tet2/3* DKO CD4 cells in the periphery even at mice immediately post weaning, at 25 days old, are more activated compared to control CD4 cells (20). Notably, mice that lack TET2 and TET3 specifically in T cells or that have a germ line deletion of *Tet2* and lack specifically *Tet3* in T cells get sick by 5 weeks old and die by 7 weeks old due to a lymphoproliferative disease (22, 25). Thus, to assess the long-term expansion potential of *Tet2/3* DKO peripheral CD4 T cells we performed serial transplantations in recipient mice (20). We discovered that *Tet2/3* DKO CD4 cells can expand in immune-competent mice and we demonstrated that expanded *Tet2/3* DKO CD4 cells exhibit increased stemness and increased genomic instability as demonstrated by increased aneuploidies (20). These expanded cells progressively downregulate CD4 expression and show decreased protein levels of the β chain of the T cell receptor (20). We have previously demonstrated that *Tet2/3* DKO iNKT cells exhibit reduced diversity of their TCRβ repertoire upon expansion (34). However, despite our systematic studies on the impact of TET proteins in conventional T cell differentiation our understanding regarding their impact on TCR expression remains elusive.

Conventional T cells express a T cell receptor (TCR) that consists of a TCRαβ heterodimer and enables them to recognize a variety of peptide antigens, spanning from self-antigens to foreign antigens-that are presented via major histocompatibility complexes (47). This broad antigen recognition is achieved through a remarkable diversity of the available TCR heterodimers (48). Each TCR chain consists of a constant region and a variable region (48). The TCRα variable region is encoded by variable (V) and joining (J) genes, whereas the TCRβ variable region is encoded by V, J and diversity (D) genes (49–51). V(D)J recombination occurs during thymic development. During this process, double strand DNA breaks occur by the recombinase complex which consists of RAG1 and RAG2 (52). RAG1 and RAG2 recognize and cleave recombination signal sequences near the V, D and J segments (53). These physiological, regulated breaks are repaired through the process of non-homologous DNA end joining (NHEJ) (51, 54, 55). First, the gene *Tcrb* that encodes the TCRβ chain is assembled through recombination events at the double negative stage (DN) 3 of thymic development (56). At this stage, the TCRβ chain associates with the pre-TCRα chain to form the pre-TCR complex (57). The DN3 stage cells that successfully assemble the *Tcrb* gene undergo β-selection, and through a proliferative burst will eventually give rise to double positive (DP) cells. This process ensures that only cells capable to form a functional TCRα β heterodimer will develop (58, 59). During β-selection *Tcrb* gene rearrangement is subject to allelic exclusion, preventing further rearrangements events of the *Tcrb* gene (60, 61). DP cells stop expressing pre-TCRα chain and start the V-J recombination at the TCR locus. The process of TCR rearrangement results in the tremendous diversity of the TCRα/β heterodimer in the surface of the T cells. The total number of TCRs that are expressed by the T cells of an individual consist their TCR repertoire. Upon antigen encounter, T cells that express TCR specific to the antigen become activated and clonally expand resulting in increased representation of T cell clones that express the same TCR. Thus, while under physiological conditions we expect a diverse TCR repertoire, in pathological conditions (infections, autoimmunity, inflammation or cancer) the TCR repertoire becomes less diverse, as specific clones become dominant (62, 63).

In this report, we compare TCRα and TCRβ repertoires at the gene expression level in control and *Tet2/3* DKO CD4 single positive (SP) cells in the thymus as well as CD4 cells isolated from the spleen of control and *Tet2/3* DKO mice at 25 days old. We focus on CD4 cells because *Tet2/3* DKO CD8 SP cells in the thymus are atypical and exhibit innate like characteristics (64). The activated phenotype of the *Tet2/3* DKO thymic CD8 SP cells is not cell-intrinsic, instead it is due to the increase of the *Tet2/3* DKO iNKT cells (22). In addition, the peripheral *Tet2/3* DKO CD8 cells are significantly reduced (22). Finally, we assess the TCR repertoire in expanded *Tet2/3* DKO T cells that acquire a stemness phenotype (20). Thus, we provide a resource that catalogues TCRα and TCRβ repertoires in CD4 SP and peripheral CD4 cells in mice. In addition, our data provides information on how concomitant loss of *Tet2* and *Tet3* impacts TCRα and TCRβ gene expression. Collectively, our data demonstrates that in our system the loss of TET2 and TET3 does not affect the diversity of TCRα and TCRβ chains during thymic development. However, upon expansion of *Tet2/3* DKO T cells in serial transplantations we observe significantly reduced diversity of both TCRα and TCRβ chains.

## Results and discussion

**Figure 1:**
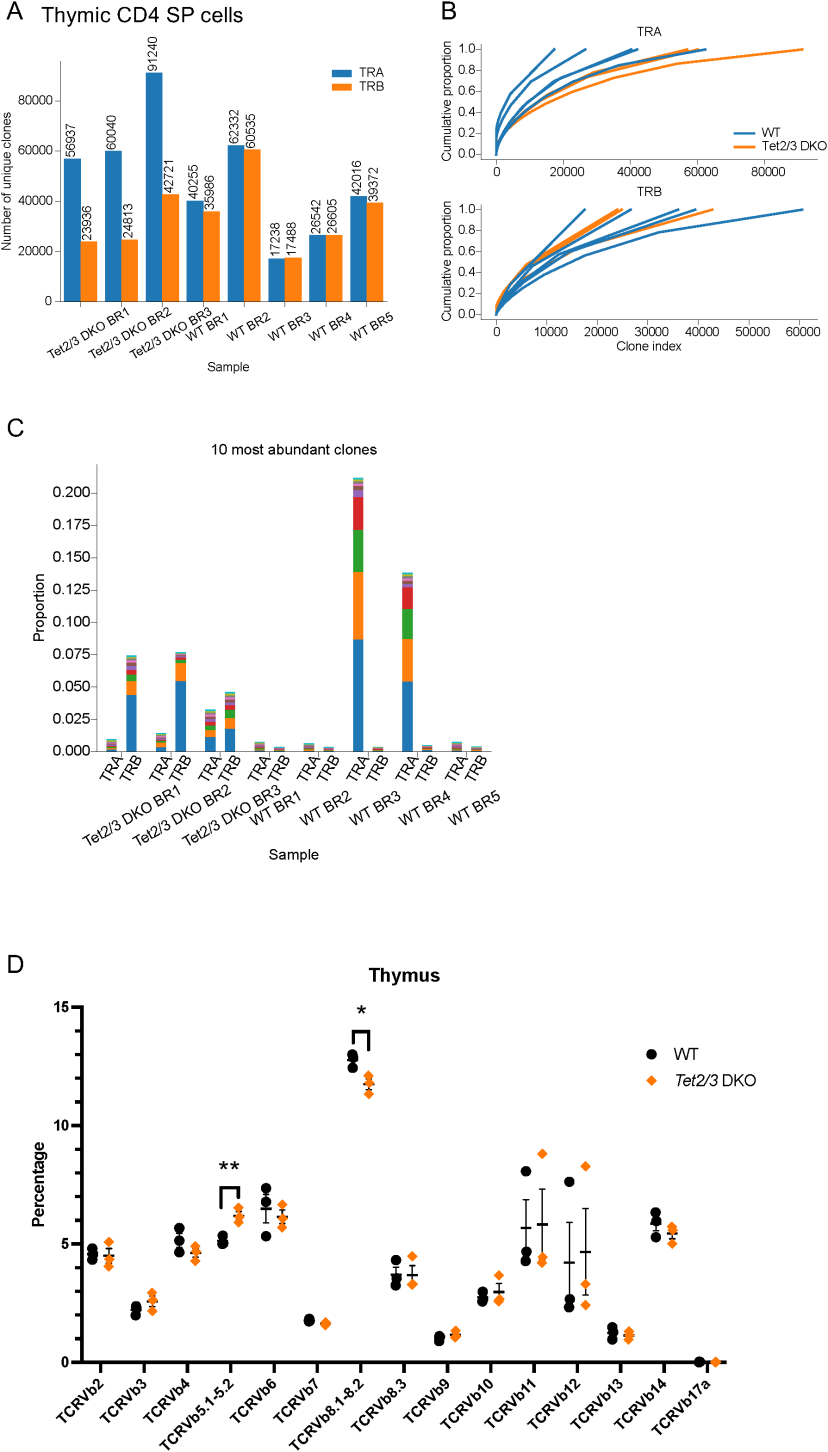
*Tet2/3* DKO thymic CD4 SP cells express similar TCRα and TCRβ chains compared to wild type CD4 SP cells. **(A)** Total RNA was isolated from wild type (WT) (n=5), and *Tet2/3* DKO (n=3) thymic CD4 SP cells (excluding aGalCer+TCRβ+ cells). TCRα (TRA, *shown in blue*) and TCRβ (TRB, *shown in orange*) repertoire was assessed by TCR SMART-seq. The numbers of identified clones per biological replicate (BR) and genotype are depicted in a bar plot. Each bar indicates the number of unique clones. **(B)** Cumulative frequency curves for TCRVα and TCRVβ chains. Each plot depicts the cumulative proportion (y axis) of the unique clones (x axis, clone index) identified by TCR SMART-seq for WT (n=5, shown in blue) and *Tet2/3* DKO (n=3, shown in orange) thymic CD4 SP cells. The slope indicates a broad distribution of clone sizes where multiple different clones contribute to the repertoire. **(C)** Bar plot indicating the proportion of the 10 most abundant clones of the TCRα and TCRβ chains as identified by TCR SMART-seq for WT (n=5) and *Tet2/3* DKO (n=3) thymic CD4 SP cells. Each clone is indicated with a different color within one sample. **(D)** Assessing TCRβ repertoire by flow cytometry. Each symbol represents a mouse. N=3 WT mice (*indicated in black dots*) and n=3 *Tet2/3* DKO mice (*shown in orange rhombus*). 3 independent experiments were performed. Unpaired student’s t-test was performed to assess statistical significance. p<0.05 (*), p<0.01 (**). Specifically, p value for TCR Vb 5.1-5.2 was 0.0076 and p value for TCR Vb 8.1-8.2 was 0.0224. Male and female mice (average age 25 days old) were evaluated.

To investigate the impact of TET proteins on the TCR repertoire in thymic murine CD4 SP cells, we depleted immature CD24 expressing thymocytes and we subsequently sorted CD4 SP cells from control and *Tet2/3 DKO* mice (**Supplementary Figure 1A**) (24, 65). We note that we excluded unconventional iNKT cells, identified as TCRβ+ aGalCer-tetramer+ cells, as we have previously described (24, 65). Then we isolated total RNA from FACS sorted CD4 SP (24) (**Supplementary Figure 1A**). For all the sorted samples the purity was above 95 percent as we have previously described (24) (**Supplementary Figures 2**, **3**). To profile the TCR repertoire using next generation sequencing, we amplified the full-length variable VJ sequences of *Tcra* transcripts and VDJ variable sequences of *Tcrb* transcripts by using TCR Switching mechanism at 5’ end of RNA template (SMART)-seq (66) and then sequenced the libraries. This approach uses as starting material total RNA and employs a 5’-Rapid amplification of cDNA ends (RACE)-based approach (66). Analysis of our datasets revealed that wild type and *Tet2/3* DKO CD4 SP cells were polyclonal and expressed diverse *Tcra* and *Tcrb* genes (**Figure 1A**, **Supplementary Tables 1**, **2**). In addition, to gain understanding on the TCR diversity across samples from another angle, we generated cumulative proportion curves (**Figure 1B**). The gradually rising curves indicate a large number of unique clones that contribute to the total TCR repertoire. Thus, our data reveals that TCR α/β libraries from wild type and *Tet2/3* DKO CD4 SP cells are highly diverse (**Figure 1B**). We next asked if there was a preference for specific clones that would be more highly expressed among samples. We identified the 10 most abundant clones in each sample for TCR α and β chain and we examined the frequency of these clones in each sample. Our analysis reveals variability both in terms of most frequent clones expressed among samples and in terms of how dominant these clones are in each sample (**Figure 1C**, **Supplementary Tables 3**, **4**). We also analyzed TCRβ repertoire at the protein level by Flow cytometry in wild type and *Tet2/3* DKO CD4 SP T cells (**Figure 1D**, **Supplementary Figures 4**, **5**). We used commercially available antibodies specific for different TCRβ chains (summarized in **Supplementary Table 5**). Our data further confirms that *Tet2/3* DKO CD4 SP cells express diverse TCRβ chains, similarly to control mice (**Figure 1D**). We note that the percentage of *Tet2/3* DKO CD4 SP cells expressing TCRVβ 8.1-8.2 chains is less compared to the control CD4 SP cells, whereas we observed an increase of TCRVβ 5.1-5.2 chains in *Tet2/3* DKO CD4 SP cells (**Figure 1C**).

**Figure 2:**
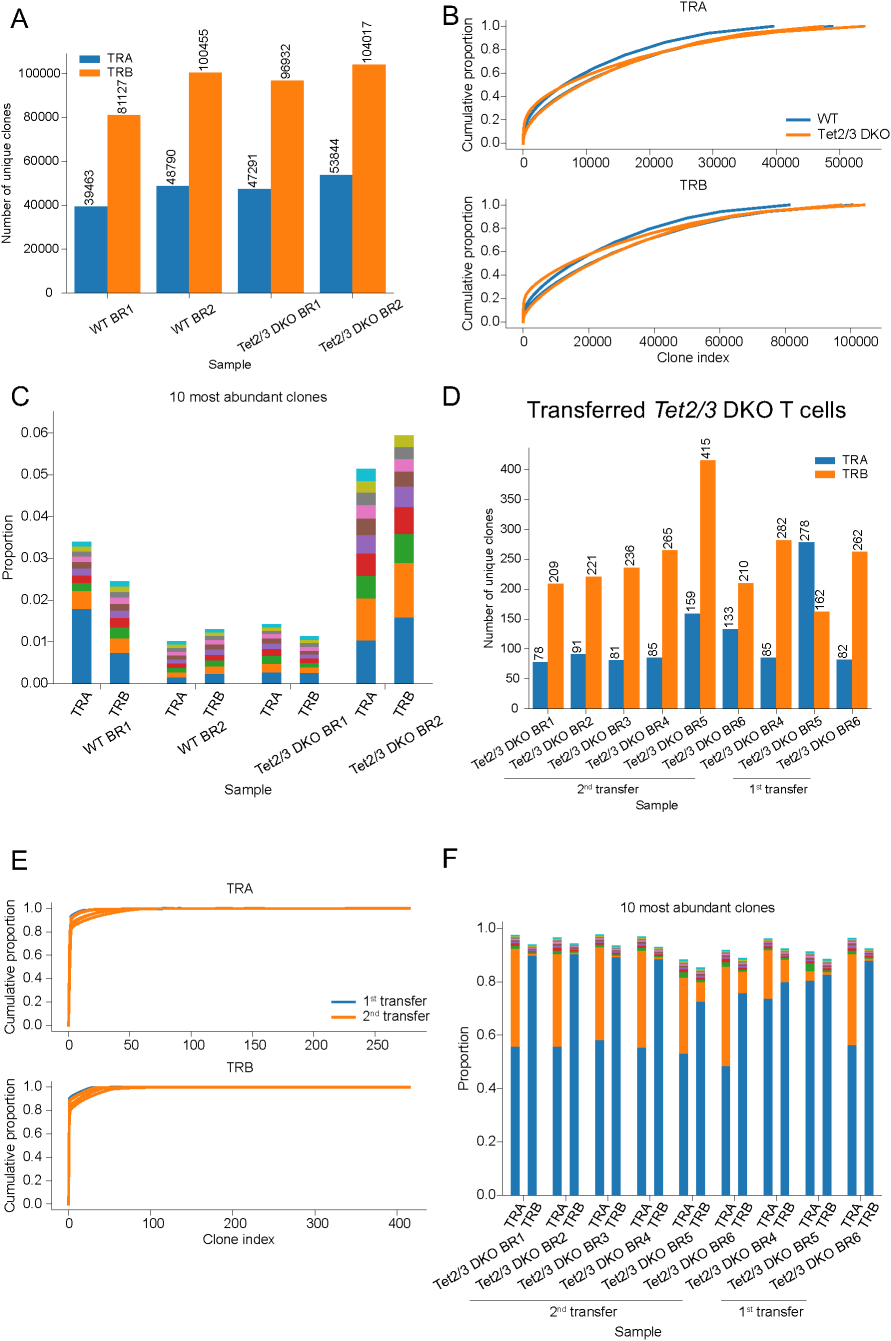
*Tet2/3* DKO CD4 T cells that have been serially transplanted in immunocompetent recipients express a limited amount of TCRα and TCRβ chains and are oligoclonal. **(A)** Total RNA was isolated from wild type (WT) total CD4 (n=2), and *Tet2/3* DKO total CD4 (n=2) cells (excluding aGalCer+TCRβ+ cells and CD25+ cells) isolated from the spleen of mice with average age 25 days old. TCRα and TCRβ repertoire was assessed by TCR SMART-seq. The numbers of identified clones per biological replicate (BR) and genotype are depicted in a bar plot. Each bar indicates the number of unique clones for TCRα (*in blue*) and TCRβ (*in orange*). **(B)** Cumulative proportion curves for TCRVα and TCRVβ chains. Each plot depicts the cumulative proportion (y axis) of the unique clones (x axis, clone index) identified by TCR SMART-seq for WT total CD4 (n=2), and *Tet2/3* DKO total CD4 (n=2) cells (excluding aGalCer+TCRβ+ cells and CD25+ cells) isolated from the spleen. **(C)** Bar plot indicating the proportion of the 10 most abundant clones of the TCRα and TCRβ chains as identified by TCR SMART-seq for WT (n=2) and *Tet2/3* DKO (n=2) CD4 cells isolated from the spleen. Different colors represent different clones within one sample. **(D)** RNA was isolated from transferred and expanded *Tet2/3* DKO CD4 cells from the spleen either after 1 transplantation (n=3) or 2 serial transplantations (n=6) in congenic recipient mice. TCRα and TCRβ repertoire was assessed by TCR SMART-seq. The numbers of identified clones per biological replicate (BR) and condition (1^st^ transfer or 2^nd^ transfer) are depicted in a bar plot. Each bar indicates the number of unique clones for TCRα (*in blue*) and TCRβ (*in orange*). **(E)** Cumulative proportion curves for TCRVα and TCRVβ chains. Each plot depicts the cumulative proportion (y axis) of the unique clones (x axis, clone index) identified by TCR SMART-seq *Tet2/3* DKO CD4 cells from the spleen either after 1 transfer (in blue, n=3) or 2 serial transfers (in orange, n=6) in congenic recipient mice. **(F)** Bar plot indicating the proportion of the 10 most abundant clones of the TCRα and TCRβ chains as identified by TCR SMART-seq for transferred and expanded *Tet2/3* DKO CD4 cells from the spleen either after 1 transfer (n=3) or 2 serial transfers (n=6) in congenic recipient mice. For all the experiments, male and female mice were evaluated.

We then proceeded to analyze TCR repertoire of peripheral CD4 T cells from young mice approximately 25 days old as we have previously described (20) (**Supplementary Figure 1B**). We depleted CD19+ B cells and we sorted total (activated and naïve) CD4 T cells, but we excluded iNKT cells and CD25 expressing cells (20) (**Supplementary Figures 6**, **7**). Sorted samples had a purity above 95% (**Supplementary Figures 6**, **7**) as previously described (20). We isolated RNA (20) and we performed TCR SMART-seq sequencing to assess TCRα and TCRβ repertoire (**Supplementary Figure 1B**). Our data indicates that wild type and *Tet2/3* DKO peripheral CD4 T cells exhibit diverse TCRα and TCRβ repertoire (**Figure 2A**, **Supplementary Tables 1**, **2**). Cumulative proportion curves further support our assessment that *Tet2/3* DKO CD4 T cells isolated from the spleen of young mice are diverse to a comparable level to wild type CD4 T cells (**Figure 2B**). We next investigated the top 10 most abundant clones for TCRα and TCRβ (**Figure 2C**, **Supplementary Tables 3**, **4**). This was confirmed for the TCRβ at the protein level by flow cytometry (**Supplementary Figure 8**). We note that while *Tet2/3* DKO CD4 cells are polyclonal they demonstrate some differences in terms of TCRβ repertoire expression (**Supplementary Figure 9**). Specifically, in the samples that we assessed, the percentage of *Tet2/3* DKO CD4 cells that express TCRVβ2 is significantly reduced whereas the percentage of TCRVβ5.1-5.2 and TCRVβ12 is significantly increased. Further research is required to address if there are functional implications for the observed differences. Another possibility is that these differences reflect stochastic variabilities in the TCR repertoire across biological replicates. Collectively, the TCRα and β repertoire is highly diverse in thymic murine CD4 SP and peripheral CD4 T cells. Regarding the *Tet2/3* DKO CD4 samples isolated either from the thymus (24) or the spleen (20) of young mice we emphasize that the sample size is relatively small. We analyzed three samples from the thymus and two from the spleen. Our data indicate that concomitant deletion of TET proteins does not compromise the TCR rearrangement and the diversity of the TCRα/β heterodimer.

We recently published that serially transplanted *Tet2/3* DKO CD4 cells exhibit upregulation of stemness genes, genomic instability and expand in the recipient mice (20). Our analysis revealed that the expanded *Tet2/3* DKO T cells demonstrated a significant and reproducible decrease at the protein level of the TCRβ expression (20). A typical characteristic of expanded, activated T cells in disease states, such as autoimmunity, inflammation and cancer, is the expansion of T cells that express a specific TCRα/β heterodimer resulting in a significant reduction of the diversity of the available TCR clones (63). To assess the TCR repertoire of the serially transplanted and expanded *Tet2/3* DKO T cells we isolated RNA from *Tet2/3* DKO T cells that have been transplanted once or twice in immunocompetent recipients as we have previously described (20) (**Supplementary Figure 1C**). The purity of the sorted cells was at least 95% (20)(**Supplementary Figure 10**). We performed TCR SMART-seq (**Supplementary Figure 1C**). The analysis revealed that the serially transplanted and expanded *Tet2/3* DKO CD4 cells expressed a very limited number of TCRα and TCRβ chains, compared to wild type CD4 cells (**Figure 2D**, **Supplementary Tables 1**, **2**). Interestingly, we did not detect a significant difference in the diversity of TCR repertoire between first and second transplantation (**Figure 2D**, **Supplementary Tables 1**, **2**). Cumulative proportion curves were very steep for the *Tet2/3* DKO samples after transfer (**Figure 2E**), indicating low diversity as a diminished number of dominant clones accounts for the TCR repertoire of these samples. These findings further support the reduced TCRα/β diversity of the *Tet2/3* DKO T cells after first and second transfer. This is in sharp contrast to the cumulative curves for the CD4 SP TCR SMART-seq data (**Figure 1B**) and the CD4 cells from the spleen of young mice TCR SMART-seq data (**Figure 2B**) where the curves were flatter, reflecting higher diversity of clones. Next, we investigated the top 10 most highly expressed clones in these samples (**Figure 2F**). In these barplots the dominant expression of one clone becomes apparent. Strikingly, in some samples the dominant clones represent more than 80% of the repertoire (**Figure 2F**, **Supplementary Tables 3**, **4**). Collectively, our analysis indicates that the oligoclonal expansion occurs during the first transplantation and that all the expanded *Tet2/3* DKO T cells can equally expand in the second transplantation in recipients with intact immune system.

**Figure 3:**
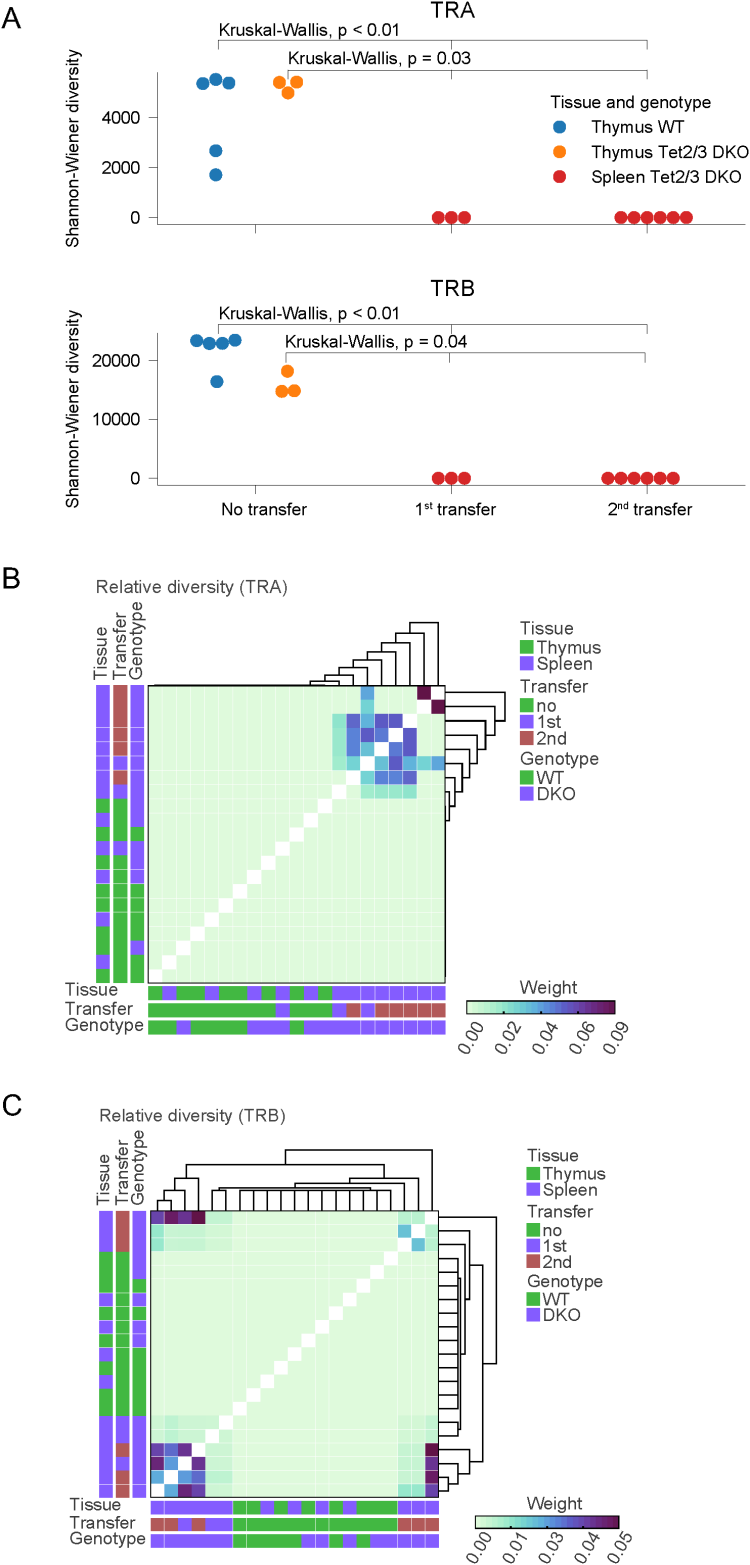
Comparing TCR diversity in control and *Tet2/3* DKO CD4 T cells during development and after transfer in congenic recipients. **(A)** Shannon-Wiener diversity comparison for TCRα and TCRβ chains determined by TCR SMART-seq for wild type (WT) (n=5) and *Tet2/3* DKO (n=3) CD4 SP samples in thymus, as well as from *Tet2/3* DKO CD4 cells that have been transferred in congenic recipients and expanded after one (n=3) or two transfers (n=6). Our analysis reveals the high diversity of the CD4 samples isolated from thymus regardless of the genotype. TCR SMART-seq for *Tet2/3* DKO CD4 cells isolated after 1^st^ or 2^nd^ transfer in congenic recipients reveals reduced diversity. Statistical analysis using the Kruskal-Wallis test reveals that *Tet2/3* DKO T cells after transfer (1^st^, 2^nd^) exhibit significantly reduced diversity compared to thymic WT CD4 SP cells (p<0.01) and compared *to Tet2/3* DKO CD4 SP cells (p=0.03). **(B)** Relative diversity was calculated between samples for the TCRα for WT CD4, *Tet2/3* DKO CD4 SP, WT CD4 isolated from spleen, *Tet2/3* DKO CD4 isolated from spleen, *Tet2/3* DKO CD4 after 1^st^ or 2^nd^ transfer in congenic recipients and plotted into heatmap. A hierarchical clustering was performed on the relative diversity weight. Parameters such as tissue (thymus/spleen), transfer (no, 1^st^, 2^nd^) and genotype (WT, DKO) are indicated. Higher weight indicates greater contribution (as measured by read counts) in the overall repertoire of the sample. The *Tet2/3* DKO samples for 1^st^ and 2^nd^ transfer have clones with high contribution in the repertoire and thus reduced diversity. **(C)** As in B, focusing on data for TCRβ.

We further analyzed our data to gain insight in the diversity of the TCR repertoire across samples. To this end we used the Shannon-Wiener index which is a function of the relative number of clonotypes present and the relative abundance of each clonotype (67). Analysis of our data revealed that *Tet2/3* DKO T cells after transfer had reduced diversity compared to WT and *Tet2/3* DKO CD4 T cells in the thymus or the spleen (**Figure 3A**, **Supplementary Figure 11**). We note that the number of distinct biological replicates varied across all the conditions from a minimum of two distinct biological replicates to a maximum of six distinct biological replicates. To perform statistical analysis of our samples we utilized the Kruskal-Wallis test, a non-parametric statistical test. Our analysis reveals that *Tet2/3* DKO CD4 T cells after first or second transfer are significantly less diverse compared to either thymic wild type or *Tet2/3* DKO CD4 SP cells (**Figure 3A**). In addition, *Tet2/3* DKO CD4 T cells after first or second transfer are significantly less diverse compared to CD4 T cells isolated from the spleen of young mice (regardless of the genotype) that have not been exposed to transfer (**Supplementary Figure 11**). Thus, the reduction of TCR diversity for both α and β chains is significant in *Tet2/3* DKO T cells after transfer. Additional comparison of the relative TCR diversity across our samples confirms that in wild type and *Tet2/3* DKO CD4 SP T cells isolated from the thymus and in wild type and *Tet2/3* DKO CD4 cells isolated from the spleen of young mice we do not observe a dominant clone, indicating high relative diversity. This observation is consistent for TCRα (**Figure 3B**) and TCRβ (**Figure 3C**) chains. On the other hand, focusing on *Tet2/3* DKO cells post transfer (first and second) it becomes apparent the presence of clones that dominate the samples and thus indicate reduced diversity (**Figures 3B, C**).

**Figure 4:**
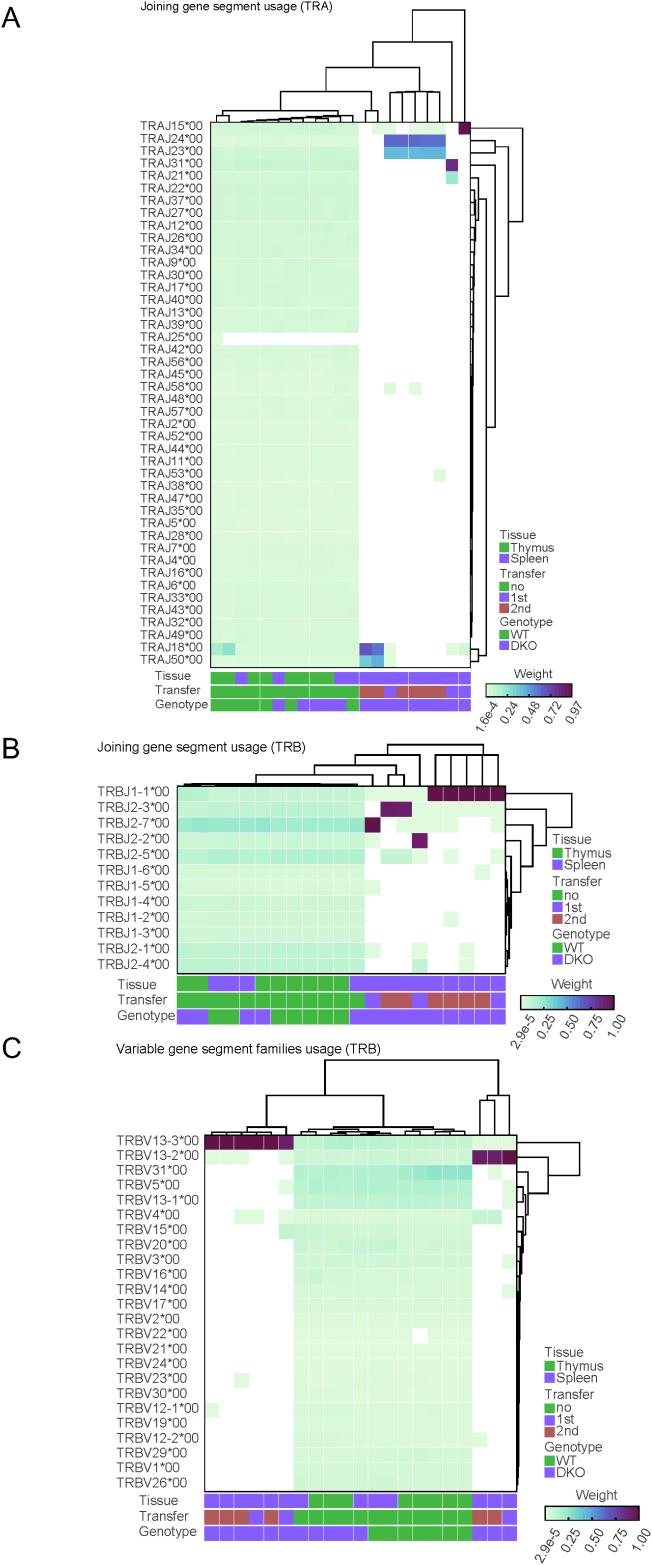
Comparison of diversity within segment usage across samples reveals reduced diversity in *Tet2/3* DKO T cells after transfer in congenic recipients. **(A)** Relative diversity was calculated between samples for the joining (J) segment gene usage for TCRα for WT CD4, *Tet2/3* DKO CD4 SP, WT CD4 isolated from spleen, *Tet2/3* DKO CD4 isolated from spleen, *Tet2/3* DKO CD4 after 1^st^ or 2^nd^ transfer in congenic recipients and plotted into heatmap. A hierarchical clustering was performed on the relative diversity weight. Parameters such as tissue (thymus/spleen), transfer (no, 1^st^, 2^nd^) and genotype (WT, DKO) are indicated. Higher weight indicates greater contribution (as measured by read counts) in the overall J gene segment usage for the TCRα repertoire of the sample. The *Tet2/3* DKO samples for 1^st^ and 2^nd^ transfer have J gene segments with high contribution in the repertoire and thus reduced diversity. **(B)** As in A, focusing on data for TCRβ. **(C)** Relative diversity was calculated between samples for the variable (V) segment gene usage for TCRβ for WT CD4, *Tet2/3* DKO CD4 SP, WT CD4 isolated from spleen, *Tet2/3* DKO CD4 isolated from spleen, *Tet2/3* DKO CD4 after 1^st^ or 2^nd^ transfer in congenic recipients and plotted into heatmap. A hierarchical clustering was performed on the relative diversity weight. Parameters such as tissue (thymus/spleen), transfer (no, 1^st^, 2^nd^) and genotype (WT, DKO) are indicated. Higher weight indicates greater contribution (as measured by read counts) in the overall V gene segment usage for the TCRβ repertoire of the sample. The *Tet2/3* DKO samples for 1^st^ and 2^nd^ transfer have J gene segments with high contribution in the repertoire and thus reduced diversity.

Our next goal was to study the diversity regarding the usage of gene segments during the V(D)J rearrangement process in our samples. Our analysis demonstrated that usage of J gene segment for TCRα (**Figure 4A**) and for TCRβ (**Figure 4B**) was diverse in CD4 cells isolated from the thymus or the spleen of wild type and *Tet2/3* DKO mice, whereas only a small subset of J segments contributed in rearrangements for TCRα (**Figure 4A**) and for TCRβ (**Figure 4B**) in *Tet2/3* DKO T cells after first or second transfer. Similar analysis for the V gene segment for TCRα (**Supplementary Figure 12**) and for TCRβ (**Figure 4C**) resulted in comparable conclusions, confirming the dominance of a small number of V gene segments that are used in *Tet2/3* DKO T cells after first or second transfer, supporting the lack of diversity in these samples.

Specifically, our analysis indicates predominant representation of *TcrVβ 13*. Interestingly, it has been reported that a patient with granular T cell leukemia had 95% dominance of TCRVβ 13.3 (68).

Our data indicates that in mice that have a germline deletion of TET2 and a deletion of TET3 at the DP cell stage using CD4-cre conditional mice (69) the TCR repertoire of conventional thymic CD4 SP is diverse. Similarly, we observe that the peripheral conventional *Tet2/3* DKO CD4 T cells are polyclonal. We must note that 5hmC and the other oxi-mCs are stable modifications, thus there are still sufficient amounts present at the DN3 stage when the VDJ recombination starts at the pre-TCRβ since at that stage only TET2 is deleted. At the DP stage when the VJ recombination occurs for the pre-TCRα, TET3 is deleted but while 5hmC levels are substantially reduced compared to wild type cells, we can still detect 5hmC (22). However, 5hmC levels are very much reduced at the thymic *Tet2/3* DKO CD4 SP cells (22). Overall, we report that in our mouse model concomitant loss of TET2 and TET3 does not appear to compromise the diversity of the TCR of CD4 cells in the thymus or in the periphery.

However, expanded *Tet2/3* DKO CD4 T cells isolated from the spleen of congenic immunocompetent recipients demonstrate reduced expression of the TCRβ compared to control CD4 cells (20). The observed oligoclonal expansion is a feature that has been previously reported in mouse models of T cell malignancy (70), including models with defects in TET2 (71). We have previously discovered that *Tet2/3* DKO CD4 T cells after transfers in congenic recipients demonstrate a stemness gene expression signature (20). Our data demonstrated that these transferred and expanded in congenic, immune-competent recipients *Tet2/3* DKO T cells could escape immune surveillance (20). We hypothesize that the dominant TCR clones potentially characterize those *Tet2/3* DKO T cells that had an increased stemness potential compared to the other clones. As a result, these clones have a proliferative advantage and preferentially expand during the transfers. We cannot exclude additional possibilities that contribute in this striking oligoclonal expansion of *Tet2/3* DKO T cells during transfers. For instance, the selected clones may exhibit increased survival compared to the other T cells that become eliminated. To formally address the above possibilities, it would be informative to perform single cell analysis to simultaneously assess TCR expression and gene expression programs in *Tet2/3* DKO CD4 T cells before transfer and at distinct time points after transfer. This approach could reveal if the clones that ultimately prevail after transfers are characterized by an enhanced stemness signature compared to the non-selected *Tet2/3* DKO CD4 T cells.

As TET proteins are tumor suppressor genes and in particular TET2 is frequently mutated in PTCL-NOS (9, 13, 72), it is important to highlight connections between our findings in murine *Tet2/3* DKO T cells (20) and human PTCL. Our samples exhibit genomic instability as reflected by increased chromosomal copy numbers (20). Indeed, aneuploidies have been reported in cases of PTCL-NOS (73). In addition, comparison of human PTCL samples reveals reduced diversity of TCR clones across the samples and clonal expansion of dominant clones, however there is no prominent TCR clone shared among all the samples potentially due to the stochastic selection of the dominant clone and the heterogeneity of the lymphomas that are grouped together in the PTCL classification (74). At the same time, we wish to emphasize that there are also differences in our model compared to human T cells that give rise to T cell lymphomas due to TET2 mutations. Specifically, in human AITL TET2 loss of function mutations co-occur with gain of function mutations of the RHOA GTPase and result in cancerous T cells that exhibit a phenotype with characteristics of follicular helper T cells (Tfh) (10, 12, 72). Detailed analysis using mouse models revealed that the gain of function mutation of RHOA was driving the Tfh skewing, whereas TET2 loss of function mutations were critical for the proliferation (75). However, our molecular and phenotypic analysis of the *Tet2/3* DKO murine T cells after transfer indicated a lack of Tfh phenotype (20). We attribute this to the fact that in the mice we use for our studies we delete simultaneously TET2 and TET3, which are the two most highly expressed TET proteins in T cells (6, 22, 24, 76). We chose this approach because our goal in our studies is to investigate the impact of profound loss of 5hmC in T cell differentiation, function and overall biology. However, we clarify that in human blood cancers, including PTCL, simultaneous mutations in TET proteins are extremely rare (73). These critical discrepancies in our study design must be carefully assessed and taken into account when we attempt to make correlations of our findings with observations relevant to human disease.

## Conclusions

In this report, we provide a resource of TCRα and TCRβ repertoire of wild type murine CD4 cells in the thymus and in spleen. In addition, we demonstrate that conventional CD4 SP cells in the thymus and peripheral CD4 cells in the spleen that lack the DNA demethylases TET2 and TET3 express a diverse TCR repertoire, with a breadth of distinct TCRα and TCRβ clones that is comparable to wild type populations. However, when we assess *Tet2/3* DKO T cells that have been serially transplanted and expanded in immunocompetent recipients we notice a striking reduction in the diversity of the TCR repertoire for both chains.

## Methods

### Mice

Mice were maintained under specific pathogen-free conditions at the Genetic Medicine building, in a facility managed by the Division of Comparative Medicine at University of North Carolina (UNC) Chapel Hill. All the experiments using mice were performed as described in our approved protocol (protocol number: 20-013, continuation protocol number: 22-252) by the UNC Institutional Animal Care and Use Committee. C57BL/6 (B6) (stock number: 000664, RRID: IMSR_JAX:000664) were obtained from Jackson Laboratories and were bred and maintained in our facility. B6. SJL-Pyprc^a^ Pepc^b^/BoyJ (common name B6 CD45.1) (stock number 002014, RRID: IMSR_JAX:002014) were purchased from Jackson Laboratories and were either used for transfers after an acclimation period or were bred and maintained in our facility. *Tet2* ^-/-^ *Tet3* ^flx/flx^ CD4Cre (22) mice have been described in previous publications. *Tet2* ^-/-^ *Tet3* ^flx/flx^ CD4Cre mice were used between 22–25 days old. For the experiments, we used sex and age-matched mice as we described (20, 24). When feasible, both male and female mice were used as indicated in the figure legends. To determine the genotype of the mutant mice we performed PCR genotyping as previously described (20, 24, 77). Mice were ear tagged and murine tissue was isolated as described in our approved mouse protocol. Genomic DNA was extracted using Phire Animal Tissue Direct PCR kit (Thermo scientific, cat no F-140WH), as described in the protocol provided by the manufacturer. To amplify DNA fragments, we performed PCR amplification using the Phire DNA polymerase (Thermo scientific) and specific primers using Biorad T100 or Biorad C1000 Touch thermocyclers. Subsequently, PCR products were run in a 3% agarose gel and were visualized using SYBR safe (Invitrogen, Thermo Scientific) staining in an Axygen Gel documentation system.

### Flow cytometry

Mice were euthanized by CO2 asphyxiation. Thymus and spleen were harvested. To prepare single-cell suspensions the organs were dissociated using a 70um cell strainer (Falcon) as previously described (65, 78). Specifically for the spleen suspension, erythrocytes were depleted using ACK lysing buffer (LifeTechnologies). Splenocytes were incubated for 10 minutes with TruStain Fc PLUS (anti-mouse CD16/32, clone: S17011E, Biolegend, Research Resource Identifier RRID: AB_2783138) at room temperature. Cells were stained in FACS buffer (PBS containing 2% FBS). Dead cells were excluded by using fixable viability dye (fixable viability dye efluor780, cat. no: 65-0865-18, eBioscience). Antibodies were used conjugated with fluorophores: CD4 APC (dilution 1:200, Biolegend, clone: RM4-5, RRID: AB_312718) or CD4 BV711 (dilution 1:200, Biolegend, clone: RM4-5, RRID: AB_11219396), CD8 PerCP-Cy5.5 (dilution 1:200, Biolegend, clone: 53-6.7, RRID: AB_2075239) or CD8 BV650 (dilution 1:200, Biolegend, clone: 53-6.7, RRID: AB_11124344), Tetramer (α-GalCer) PE (dilution 1:400, from NIH tetramer core), B220 PE-Cy7 (dilution 1:400, Biolegend, clone: RA3-6B2, RRID: AB_313004). To assess TCRβ repertoire, cells were also stained using the β TCR Screening Panel FITC (BD Pharmingen). For each clone, 20 µL of the corresponding antibody were added per reaction, according to manufacturer instructions (**Supplementary Table 5**). aGalactosyl-ceramide loaded tetramer conjugated with PE was obtained from NIH tetramer Core and was used in a dilution 1:400 per staining reaction.

Samples were analyzed by Flow Cytometry in a Novocyte 3005 (ACEA, Agilent). For data acquisition the NovoExpress software (Agilent) was used. Data analysis and FACS plots generation was performed using FlowJo (Treestar).

### Cell enrichment & flow cytometry activated sorting

The detailed procedures for enriching, sorting and obtaining thymic *WT* and *Tet2/3* DKO CD4 SP cells, WT and *Tet2/3* DKO CD4 cells from the spleens and *Tet2/3* DKO expanded cells have been previously described (20, 24). Briefly, thymocytes were dissociated as previously described (65, 78) and were enriched for mature T cell subsets by depleting CD24+ cells, using biotinylated anti-mouse CD24 (clone:M1/69, RRID: AB_312837, Biolegend) (24). Splenocytes were treated with ACK buffer (Life Technologies) to remove erythrocytes and were depleted for B cells using biotinylated anti-mouse CD19 (clone: 6D5, RRID: AB_313639), any remaining blood cells using biotinylated anti-mouse TER119/Erythroid cells (clone: TER119, RRID: AB_313705). Cells were stained with CD4 AF488 (clone: RM4-5, RRID: AB_389303), TCRβ PERCPCy5.5 (clone: H57-597, RRID: AB_1575173), CD25 APC (clone: PC61, RRID: AB_312861), aGalactocyl-Ceramide (aGal-Cer) loaded Tetramer PE. Live CD4^+^, CD25^-^, TCRβ^+^, tetramer- cells were sorted and used in downstream applications. Expanded cells in congenic recipients were sorted after staining with CD45.1 BV421 (clone: A20, RRID: AB_2562563) and CD45.2 PE (clone: 104, RRID: AB_313445) from Biolegend. We have previously described the sorting strategy and we have evaluated the purity of the sorted samples that were used to isolate RNA (20, 24). Our sorted samples have a minimum purity of 95% (20, 24) (**Supplementary Figures 2**, **3**, **6**, **7**, **10**). Part of this RNA was used for the TCR repertoire analysis using TCR sequencing for this study.

In all cases, dead cells were excluded using a fixable viability dye eFluor780 (eBioscience).

The cells were sorted using an Aria Sorter (Becton Dickinson).

### Adoptive transfer of *Tet2/3* DKO CD4 cells in congenic recipient mice

The serial transplantation and expansion experiments of *Tet2/3* DKO CD4 T cells in CD45.1 non-irradiated, immune-competent mice were described (20). *Tet2/3* DKO T cells for the first and the second transplantation were isolated and characterized in the context of our previous study (20).

### RNA isolation

RNA used in this study has been isolated from murine samples that were analyzed and described in our previous studies (20, 24). To isolate RNA from sorted subsets we used the RNA plus mini kit (Qiagen, catalogue number: 74134) or RNA plus micro kit (Qiagen, catalogue number: 74034) depending on the starting material as we have previously described (20, 24, 65, 77). Typically for expanded *Tet2/3* DKO T cells we had a starting material of 5 million cells and we used the RNA plus mini kit, whereas for wild type and *Tet2/3* DKO thymic or peripheral CD4 T cells we used a maximum of 600.000 cells and we isolated cells with RNA plus micro kit. In all cases we followed the instructions provided in the accompanying protocols by Qiagen. RNA quantity was determined using High Sensitivity RNA kit in a Qubit 4 Fluorometer (Thermo Fischer). RNA integrity was evaluated using Tapestation or Bioanalyzer (Agilent). All the samples had an RNA integrity number (RIN) value above 9.

### TCR-sequencing

In order to analyze TCR repertoire, we used the SMARTer mouse TCR α/β profiling kit (Takara, catalogue number 634403) following the manufacturer’s instructions. We used 20 ng of total RNA (isolated as described above) in order to synthesize cDNA for each library. The kit employs a 5’-RACE-based approach to capture complete V(D)J variable regions of TCR transcripts. The libraries were sequenced using a Miseq sequencer (Illumina) at the UNC High Throughput Sequencing Facility (HTSF) (*data shown in* **Figures 1B**, **3B**) or the Duke University School of Medicine for the use of Sequencing and Genomic Technologies Shared Resource (*data shown in* **Figure 2B**). In this study we analyzed a total of 21 libraries (**Supplementary Table 6**).

### Statistics and reproducibility

Data were analyzed using Prism software (Graphpad). Unpaired student’s *t* test was applied as indicated. In each figure legend, the indicated *P*-values are described. Data are mean ± s.e.m. In the graphs, each dot represents a mouse. For statistical analysis of the Shannon-Wienner diversity across different samples we used the Kruskal-Wallis test. Unless otherwise indicated the *P* value was not statistically significant (*P* > 0.05). Differences were considered significant when p < 0.05 (^∗^); < 0.01 (^∗∗^); < 0.001 (^∗∗∗^).

For the phenotyping experiments, age-matched mice from different litters and of different sex were evaluated, with reproducible results. In addition, we ensured that a minimum of 2 independent experiments was performed in each case.

### TCR-seq data analysis

Adapter trimming and quality filtering of the sequencing libraries was done using fastp (0.21.0) (79) with the default parameters. The TCR repertoire extraction (–receptor-type tcr) was done using MiXCR (v4.7.0) (80) was used was used to assess TCR repertoire from TCR-seq data using the takara-mouse-rna-tcr-smarter preset (mixcr analyze takara-mouse-rna-tcr-smarter). MiXCR was used to assess the quality of the samples (mixcr exportQc align *.vdjca alignQC.pdf). MiXCR was used to run postanalysis routines (mixcr postanalysis individual –metadata metadata.tsv –default-downsampling count-read-auto –default-weight-function read *.clns result.json.gz and mixcr postanalysis overlap –metadata metadata.tsv –default-downsampling count-read-auto –default-weight-function read *.clns overlap.json.gz). MiXCR was used to assess V and J gene usage (mixcr exportPlots vUsage result.json.gz vusage.pdf and mixcr exportPlots jUsage result.json.gz vusage.pdf). MiXCR was used to visualize Shannon-Wiener diversity (mixcr exportPlots diversity –metric shannonWiener) and relative diversity (mixcr exportPlots overlap –metric RelativeDiversity). All the libraries were complex with a range of duplication rate ranging between 0.0005 to 0.0010 (**Supplementary Figure 13A, B**). Comparison of the mappable reads across the samples revealed variability. Specifically, reads for all the TCR SMART-seq libraries generated from WT and *Tet2/3* DKO CD4 SP thymic cells or peripheral CD4 T cells were approximately 55% successfully aligned (**Supplementary Figure 13C**). However, reads obtained for the TCR SMART-seq libraries for *Tet2/3* DKO T cells varied from 30% to 80% successful alignment to the reference genome (**Supplementary Figure 13**). Such variability has been previously reported for TCR-seq libraries prepared using 5-RACE approach (81, 82). From a technical standpoint, the lower alignment efficiency can be attributed to relatively increased number of shorter fragments (82). However, it becomes evident that from all our samples the lower alignment is observed specifically to some libraries using RNA from transferred *Tet2/3* DKO T cells that have proliferated to dominate the spleen of the recipient mice (20). While the RNA integrity of the isolated RNA was at least 9 (20) we believe that the downregulation of the TCR observed in these activated samples and the overall compromised health status of the recipient mice may have contributed in the reduced efficiency of alignment consistent with previous reports for samples with malignant traits (81).

## Data Availability

TCR-seq datasets have been deposited in the Gene Expression Omnibus (GEO) public repository under the accession code GSE190231 (https://www.ncbi.nlm.nih.gov/geo/query/acc.cgi?acc=GSE190231).

## Supplementary material

Supplementary Table 1 Summary of *Tcra* sequences identified by TCR-seq. The source table is deposited in Figshare. https://doi.org/10.6084/m9.figshare.29293649.v1.

Supplementary Table 2 Summary of *Tcrb* sequences identified by TCR-seq. The source table is deposited in Figshare. https://doi.org/10.6084/m9.figshare.29640680.v1.

Supplementary Table 3 Summary of top 10 TCRα clones observed in the examined samples in this study.

Supplementary Table 4 Summary of top 10 TCRβ clones observed in the examined samples in this study.

Supplementary Table 5 Summary of the antibodies used to assess TCRβ clones by flow cytometry. This table is provided in a combined pdf as supplementary material.

Supplementary Table 6 Sequencing metrics. Table summarizing the number of reads for each of the sequenced libraries. This table is provided in a combined pdf as supplementary material. Supplementary Figures 1–13 and their accompanying legends are provided in a combined pdf file as supplementary material.
