# Supplementary material for: Assessing the impact of TET2 and TET3 deletion in TCRalpha and TCRbeta repertoire in murine CD4 T cells in physiological and pathophysiological conditions
Source: Front Immunol. 2025 Aug 20;16:1638500. doi: 10.3389/fimmu.2025.1638500 (PMC12405334; doi:10.3389/fimmu.2025.1638500)
Supplement: Supplementary file 1 [file DataSheet1.pdf]

Supplementary Table 5 : Antibodies in the TCRβ repertoire screening panel

β TCR Screening Panel FITC	Fluorophore	Company BD Pharmingen	Clone	Catalogue number 557004
TCRb clone	Fluorophore			
Vβ2	FITC	BD Pharmingen	B20.6	51-01634L
Vβ3	FITC	BD Pharmingen	KJ25	51-01404L
Vβ4	FITC	BD Pharmingen	KT4	51-01934L
Vβ5.1-5.2	FITC	BD Pharmingen	MR9-4	51-01354L
Vβ6	FITC	BD Pharmingen	RR4-7	51-01364L
Vβ7	FITC	BD Pharmingen	TR310	51-01424L
Vβ8.1-8.2	FITC	BD Pharmingen	MR5-2	51-01344l
Vβ8.3	FITC	BD Pharmingen	1B3.3	51-09044L
Vβ9	FITC	BD Pharmingen	MR10-2	51-01384L
Vβ10b	FITC	BD Pharmingen	B21.5	51-01644L
Vβ11	FITC	BD Pharmingen	RR3-15	51-01374L
Vβ12	FITC	BD Pharmingen	MR11-1	51-01684L
Vβ13	FITC	BD Pharmingen	MR12-3	51-01394L
Vβ14	FITC	BD Pharmingen	14-2	51-01564L
Vb17a	FITC	BD Pharmingen	KJ23	51-01414L

Supplementary Table 6: Sample and number of reads

Library	Sample	Description	Reads	Aligned Reads
TSAGLAB1	splj3_6	2ndary transfer	720694	305898
TSAGLAB3	splj8_1	1st transfer	856158	348448
TSAGLAB4	splj13_1	1st transfer	1954720	472645
TSAGLAB5	splj15_2	1st transfer	1973234	355087
TSAGLAB6	splj22_1	2ndary transfer	1508836	263032
TSAGLAB7	splj22_2	2ndary transfer	1917702	318524
TSAGLAB8	splj22_3	2ndary transfer	2132338	365273
TSAGLAB9	splj22_4	2ndary transfer	2138926	374108
TSAGLAB10	wt1_thy	CD4 cells from WT thymus	1465704	427131
TSAGLAB11	wt2_thy	CD4 cells from WT thymus	3132254	901889
TSAGLAB12	wt3_thy	CD4 cells from WT thymus	647900	189742
TSAGLAB13	wt4_thy	CD4 cells from WT thymus	951192	283630
TSAGLAB14	wt5_thy	CD4 cells from WT thymus	1780578	508955
TSAGLAB19	j3-5	2nd transfer	2019900	861439
TSAGLAB20	thy b39-1	CD4 cells from thymus of Tet2/3 DKO	1479428	471312
TSAGLAB21	thy b56-1	CD4 cells from thymus of Tet2/3 DKO	1698750	526469
TSAGLAB22	thy b56-6	CD4 cells from thymus of Tet2/3 DKO	2966312	936322
B34-1	spl B34-1	CD4 cells from spleen of Tet2/3 DKO	7358562	2145605
B39-1	spl B39-1	CD4 cells from spleen of Tet2/3 DKO	5317034	1669310
WT2	wt2_spl	CD4 cells from spleen of WT	5471980	1670306
WT3	wt3_spl	CD4 cells from spleen of WT	6020352	1865670

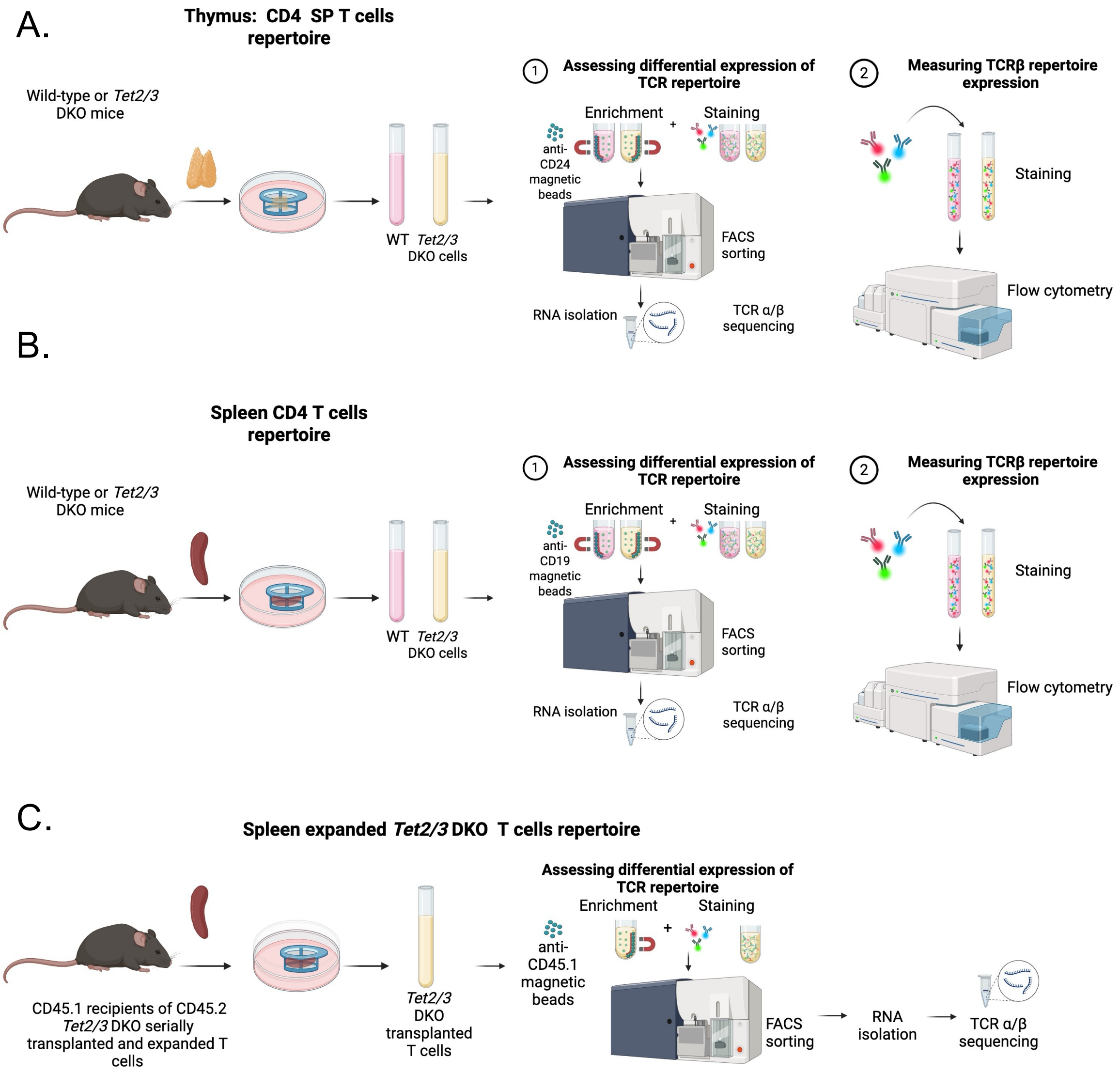

Supplementary Figure 1: Experimental outline. Strategy for assessing TCR repertoire in **A.** CD4 SP thymic T cells isolated from wild type or *Tet2/3* KO mice, **B**. CD4 cells isolated from the spleen of wild type or *Tet2/3* DKO mice, **C.** from *Tet2/3* DKO CD4 T cells after transfer to congenic recipients. This figure was created with Biorender.

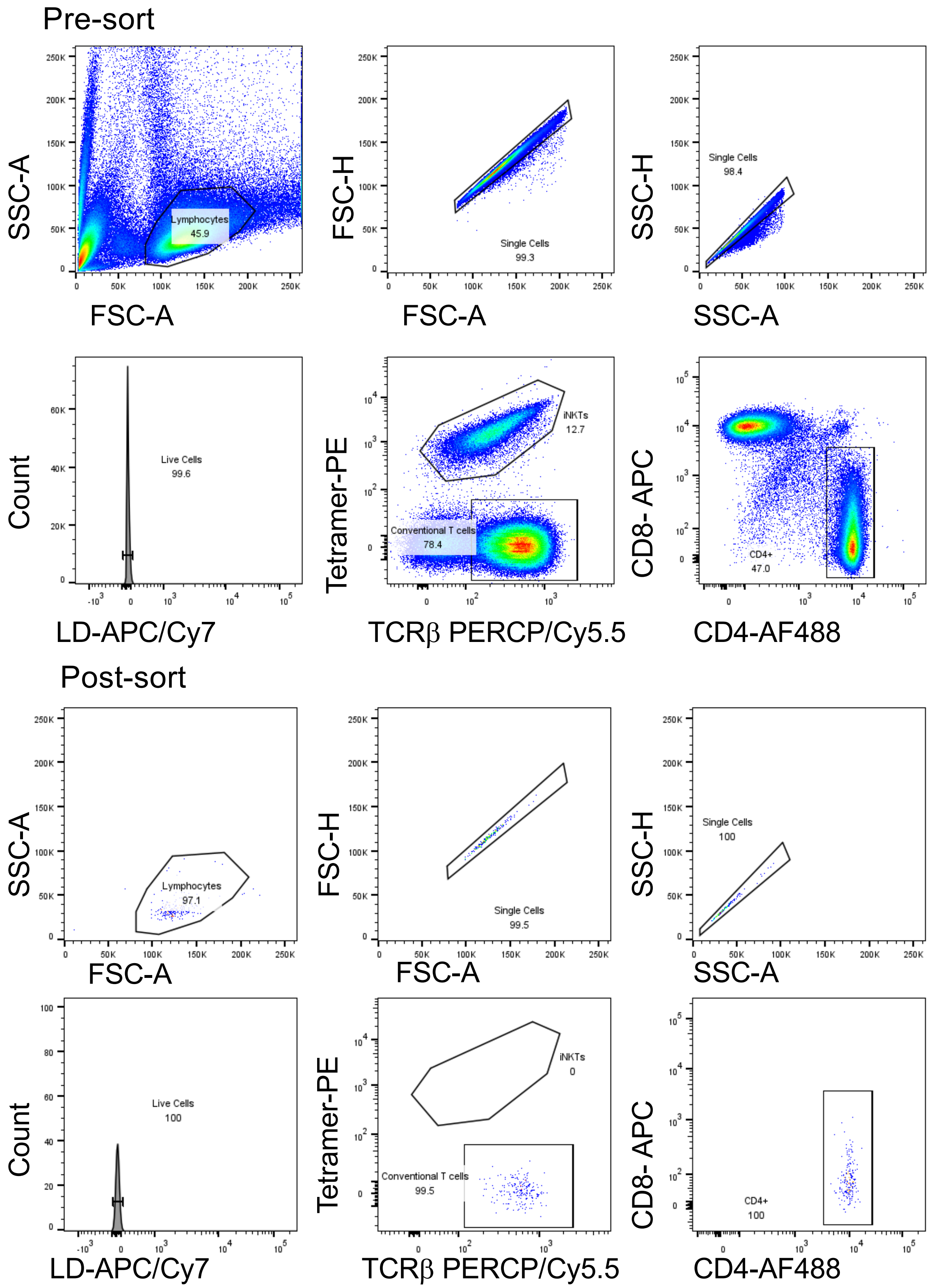

Supplementary Figure 2: Sorting strategy to isolate WT thymic CD4 SP T cells.

Pre-sort: Gating strategy to isolate WT CD4 SP T cells from thymus. Forward scatter (FSC) and side scatter (SSC) were plotted and gating for lymphocytes is depicted. Singlets were gated and doublets were excluded based on forward and side scatter plots. Dead cells staining positive for a live/ dead (LD) exclusion dye were excluded and live cells (negative for the live/dead dye) were gated. iNKT cells (tetramer+ and TCRβ intermediate) were excluded. CD4 positive cells were gated and sorted. CD8 cells are also depicted. **Post-sort**: After sorting, a small quantity of the sample was assessed for purity.

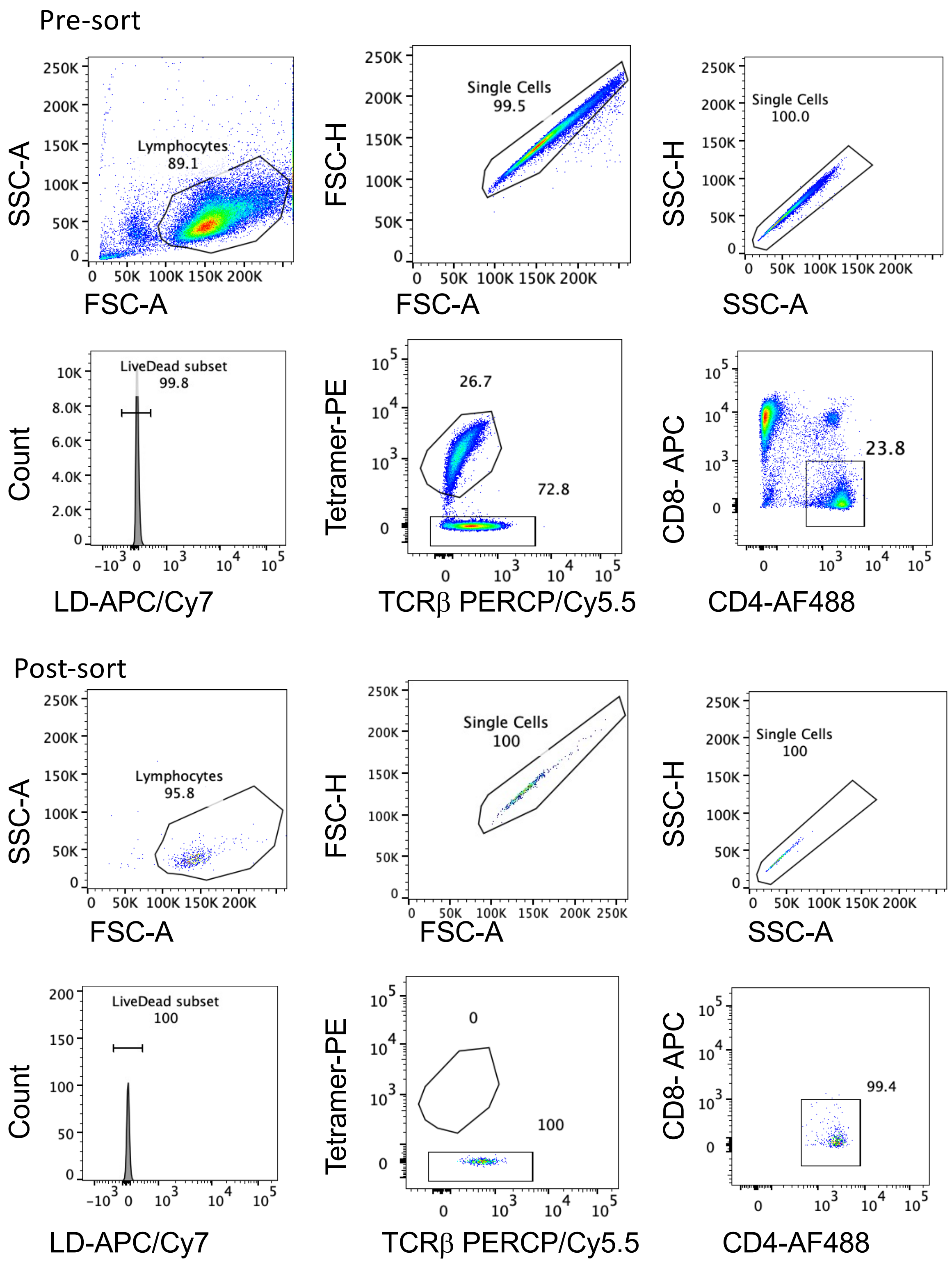

Supplementary Figure 3: Sorting strategy to isolate *Tet2/3* DKO thymic CD4 SP T cells. Pre-sort: Forward scatter (FSC) and side scatter (SSC) were plotted. Lymphocytes were gated. Singlets were gated and doublets were excluded based on forward and side scatter plots. Dead cells that stained positive for a live/ dead exclusion dye were excluded and live cells (negative for the live/dead dye) were gated. iNKT cells (tetramer+ and TCRβ intermediate) were excluded. CD4 positive cells were gated and sorted. CD8 cells are also depicted.

Post-sort: After sorting, a small quantity of the sample was assessed for purity.

Wild type thymic CD4 SP

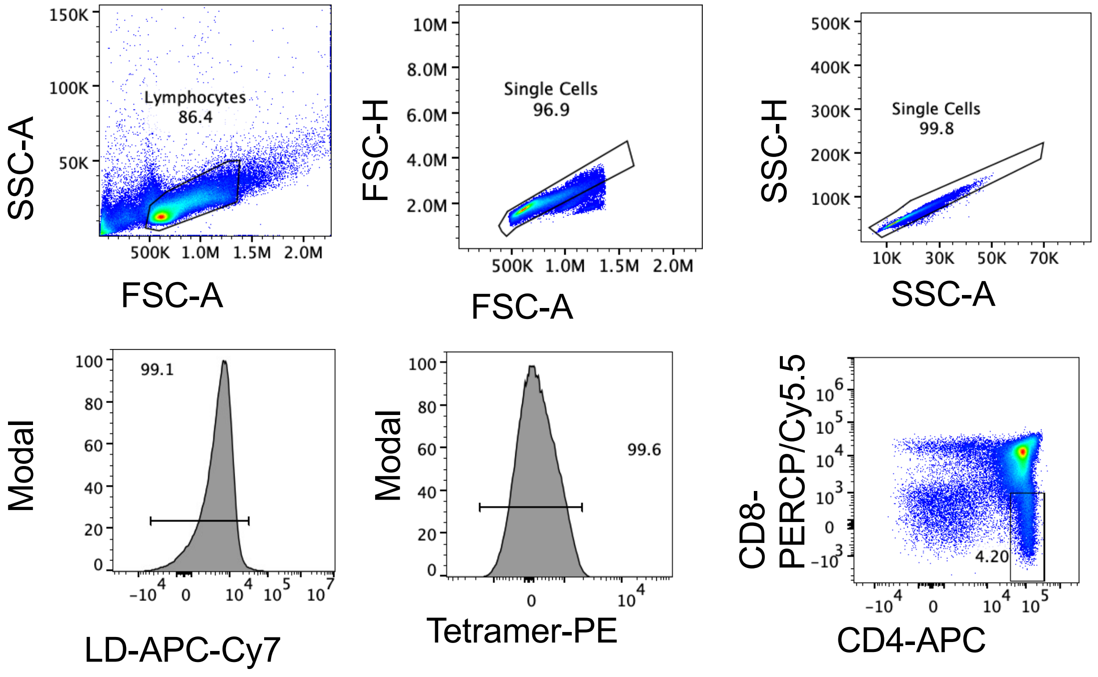

Tet2/3 DKO thymic CD4 SP

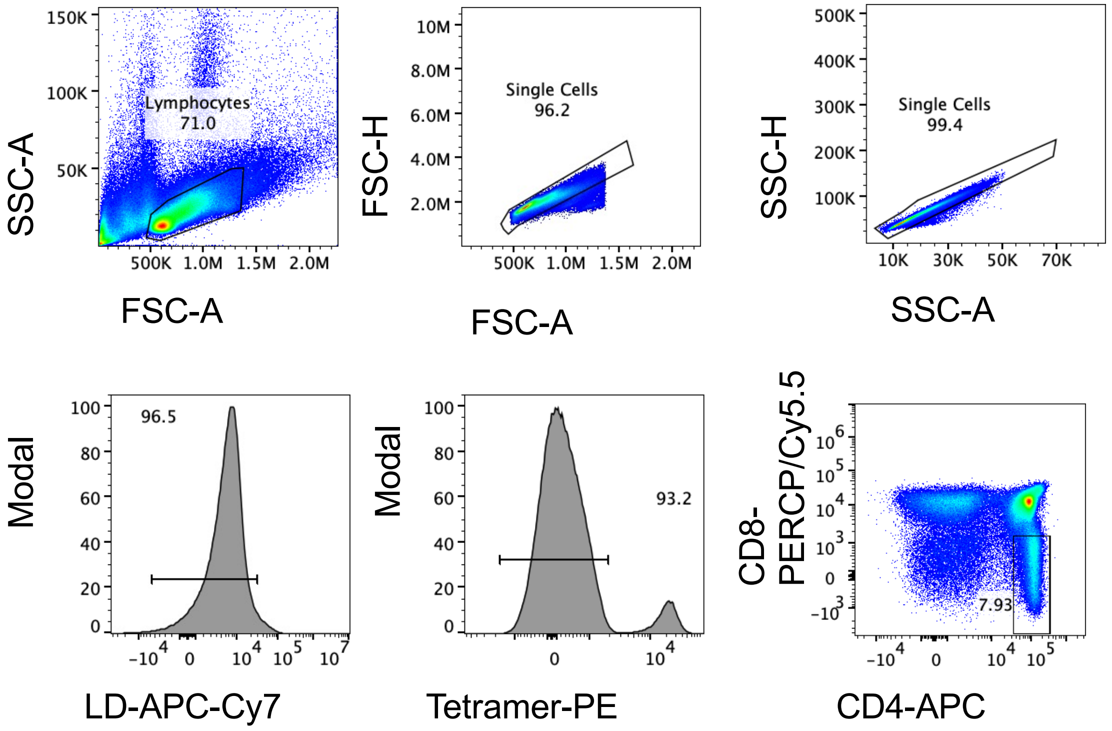

Supplementary Figure 4: Gating strategy for thymic CD4 SP cells relevant to figure 1 D. Gating strategy for thymic CD4 SP cells in wild type or *Tet2/3* DKO mice is shown. One representative experiment out of three independent experiments is depicted.

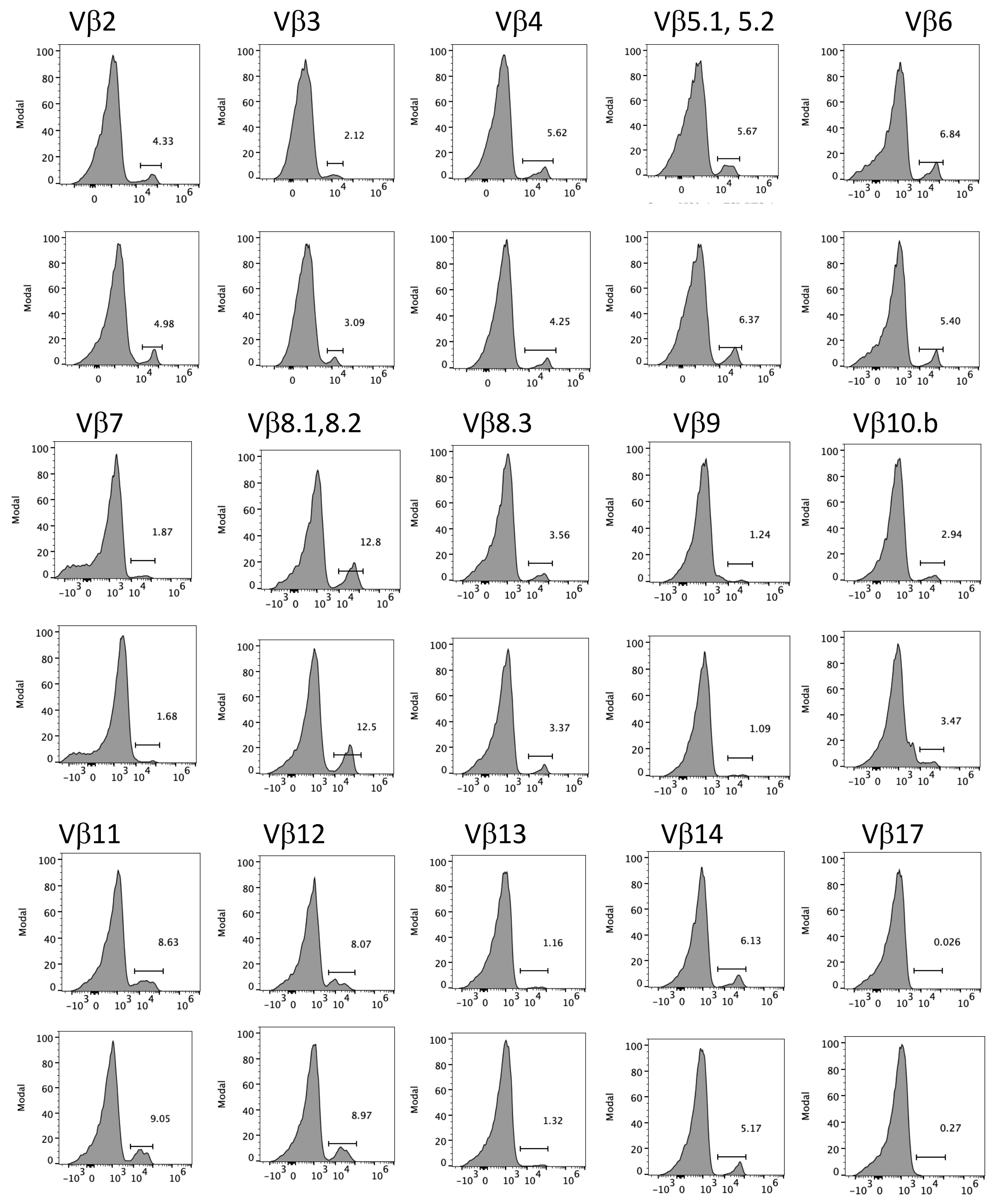

Supplementary Figure 5: Gating strategy for TCR Vβ in thymic CD4 SP T cells relevant to Figure 1D. Histograms depicting the expression of different TCRβ clones assessed by flow cytometry in wild type (upper panel for each clone) or *Tet2/3* DKO (lower panel for each clone) CD4 SP T cells. Results from one representative experiment are shown. Three independent experiments were performed.

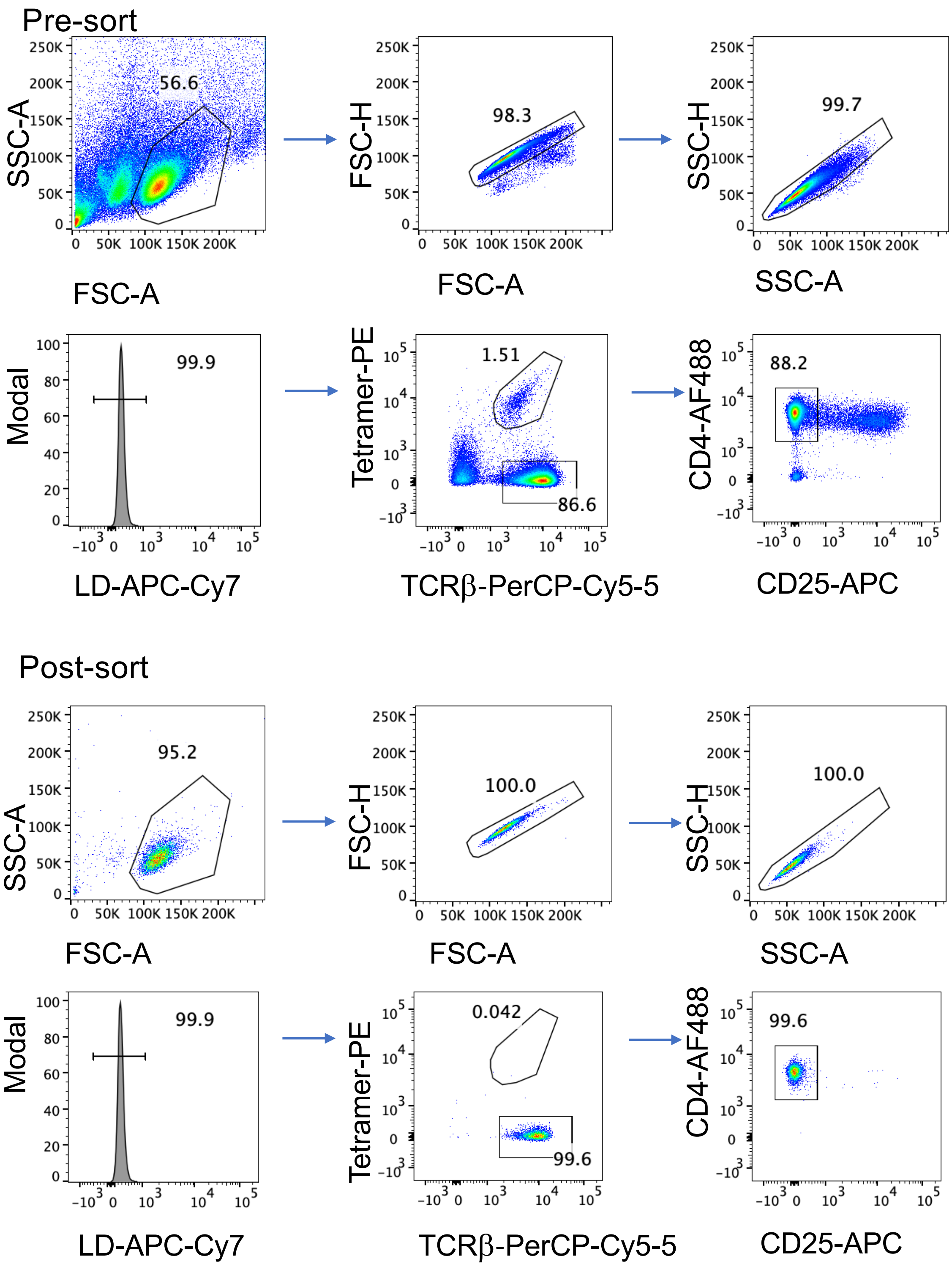

Supplementary Figure 6: Sorting strategy to isolate CD4 cells from the spleen of wild type mice. Pre-sort: Forward scatter (FSC) and side scatter (SSC) were plotted. Lymphocytes were gated. Singlets were gated and doublets were excluded based on forward and side scatter plots. Dead cells that stained positive for a live/ dead (LD) exclusion dye were excluded and live cells (negative for the live/dead dye) were gated. iNKT cells (tetramer+ and TCRβ intermediate) were excluded. CD4 positive, CD25 negative cells were gated and sorted. **Post-sort**: After sorting, a small quantity of the sample was assessed for purity.

Figure from Gioulbasani, Äijö et al, Communications Biology, 2024

PMID: 39627458, Creative Commons CC-BY-NC-ND

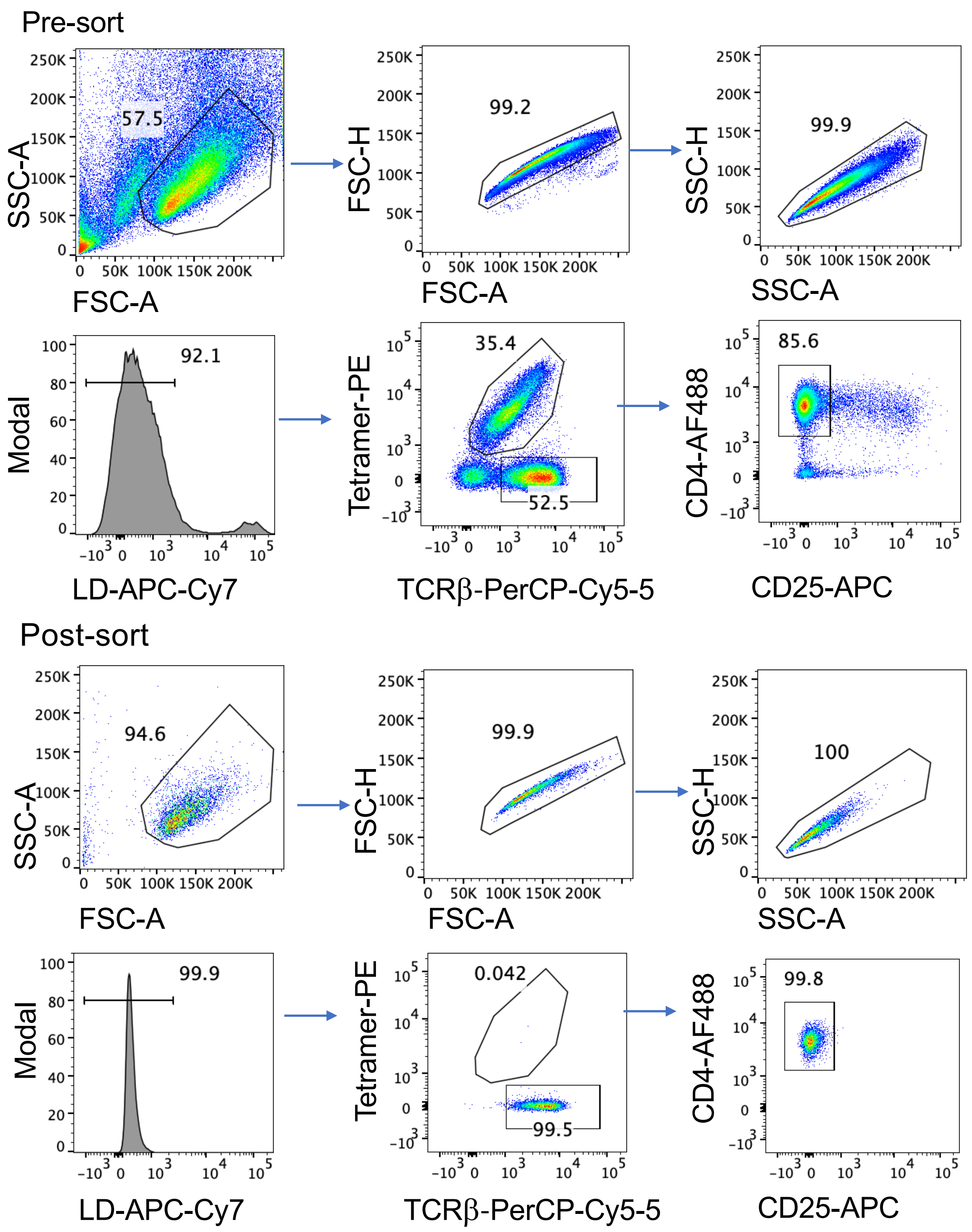

Supplementary Figure 7: Sorting strategy to isolate CD4 cells from the spleen of *Tet2/3* DKO mice. Pre-sort: Forward scatter (FSC) and side scatter (SSC) were plotted. Lymphocytes were gated. Singlets were gated and doublets were excluded based on forward and side scatter plots. Dead cells that stained positive for a live/dead (LD) exclusion dye were excluded and live cells (negative for the live/dead dye) were gated. iNKT cells (tetramer+ and TCRβ intermediate) were excluded. CD4 positive, CD25 negative cells were gated and sorted.

Post-sort: After sorting, a small quantity of the sample was assessed for purity.

Figure from Gioulbasani, Äijö et al, Communications Biology, 2024.

PMID: 39627458, Creative Commons CC-BY-NC-ND

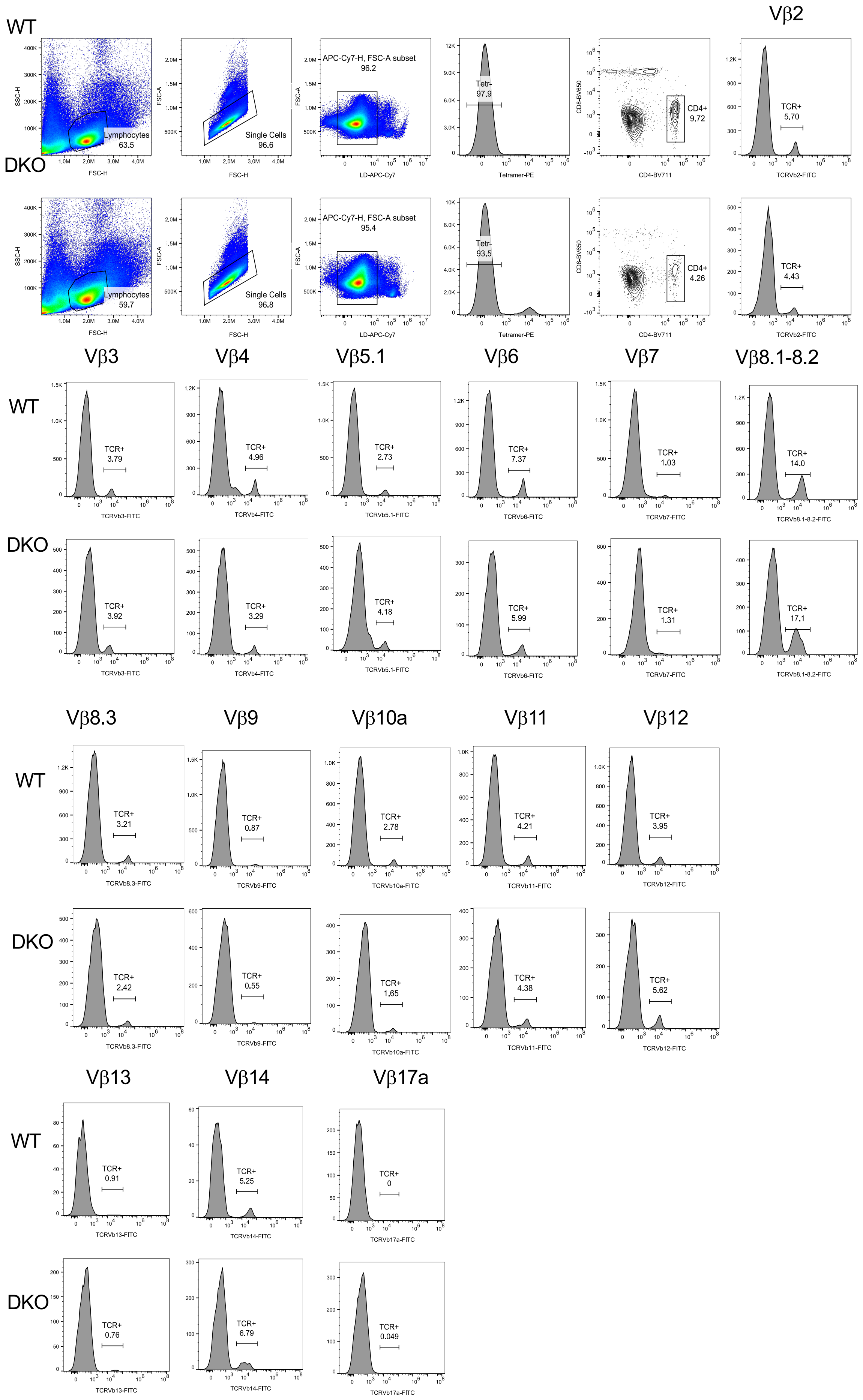

Supplementary Figure 8

Supplementary Figure 8: Gating strategy to identify TCRVβ clones expressed in WT and *Tet2/3* DKO CD4 T cells in spleen.

Gating strategy for CD4 cells in spleens isolated from wild type or *Tet2/3* DKO mice is shown. Histograms depicting the expression of different TCRβ clones assessed by flow cytometry in wild type (upper panel for each clone) or *Tet2/3* DKO (lower panel for each clone) CD4 T cells. Results from one representative experiment are shown. Four independent experiments were performed.

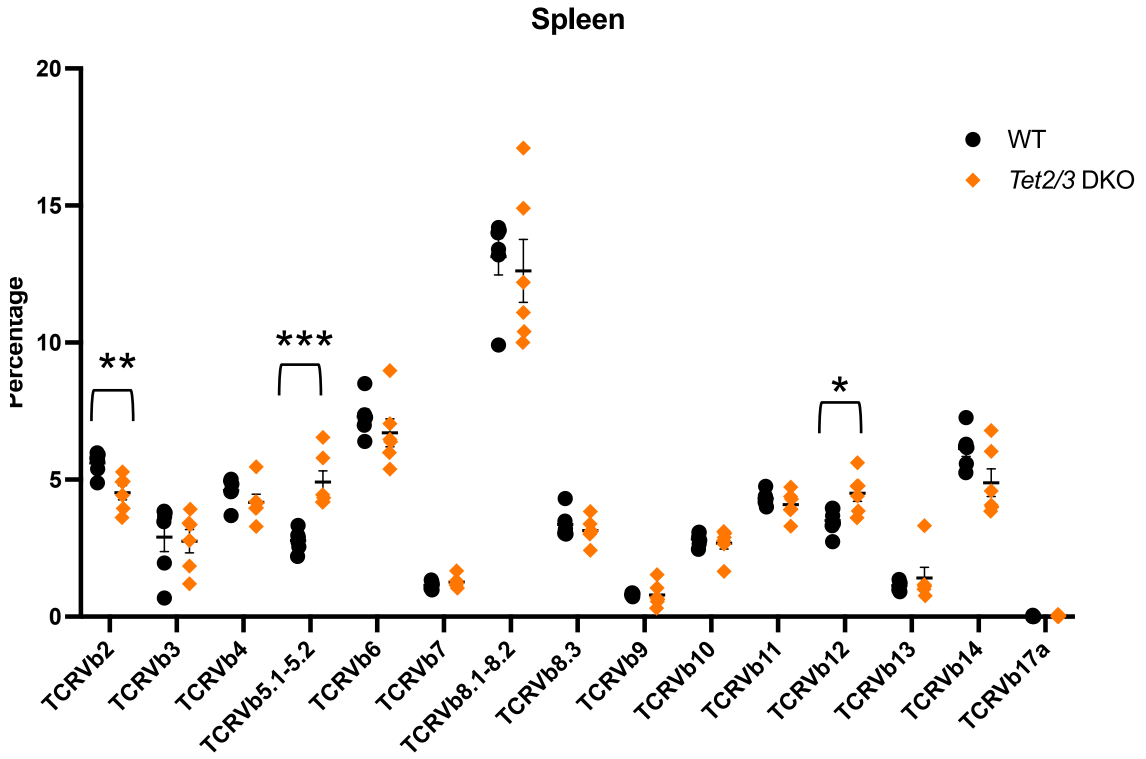

Supplementary Figure 9 *: Tet2/3* DKO peripheral CD4 cells express similar TCR β chains compared to wild type CD4 T cells

TCRβ repertoire at the protein level was evaluated by Flow cytometry. Each symbol represents a mouse. N=5 WT mice (*indicated in black dots*) and n=5 *Tet2/3* DKO mice (*shown in orange rhombus*) of an average age of 25 days old were assessed. 4 independent experiments were performed. Male and female mice were evaluated. Unpaired student's t-test was performed to assess statistical significance. $p<0.05$ (*), $p<0.01$ (**), $p<0.001$ (***). Specifically, p value for TCR Vβ 2 was p=0.0058, for TCR Vβ 5.1-5.2 was p=0.0007 and p value for TCR Vβ 12 was 0.0174.

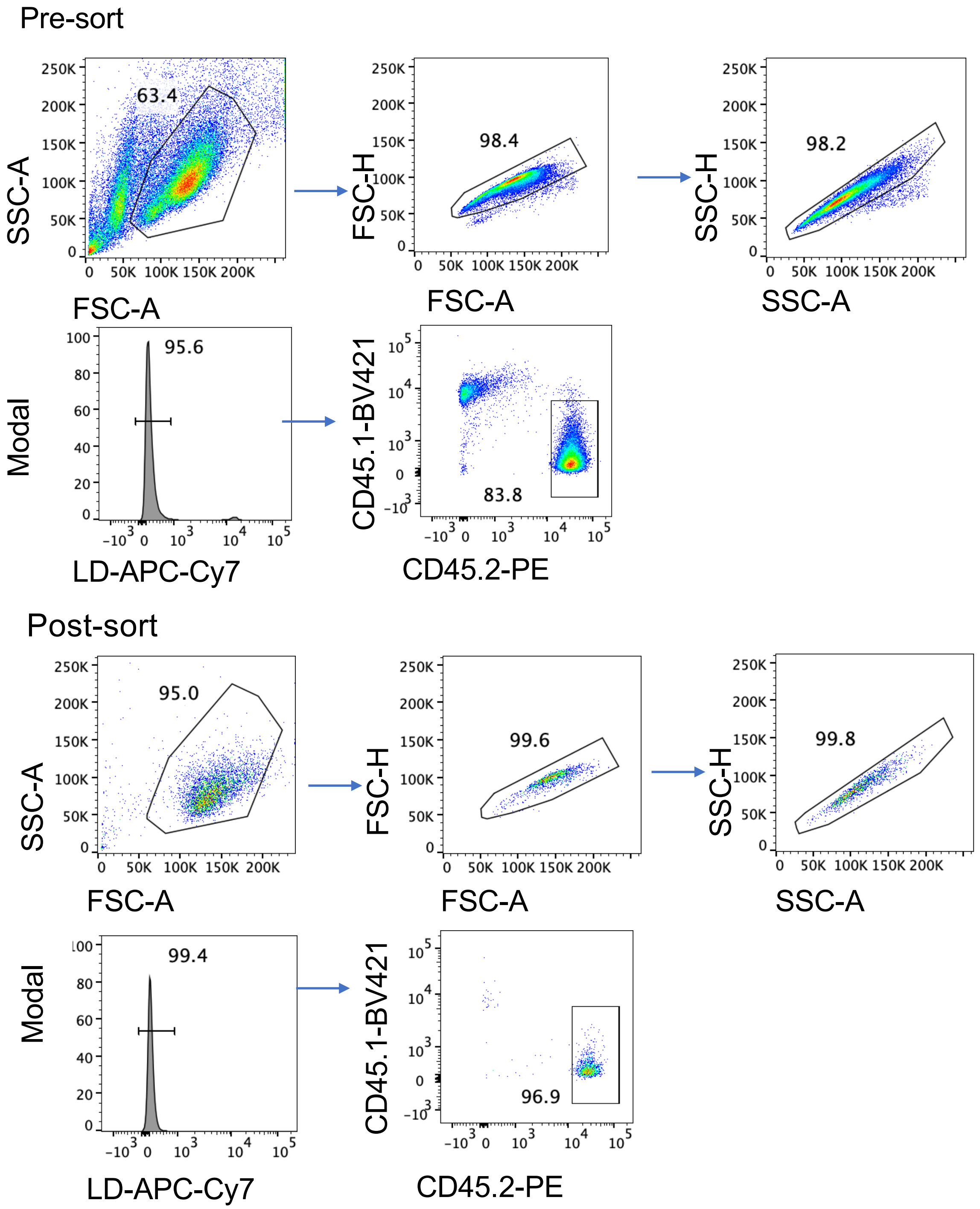

Supplementary Figure 10: Sorting strategy to isolate CD45.2+ *Tet2/3* DKO T cells after transfer in CD45.1+ recipients. Pre-sort: Forward scatter (FSC) and side scatter (SSC) were plotted. Lymphocytes were gated. Singlets were gated and doublets were excluded based on forward and side scatter plots. Dead cells that stained positive for a live/ dead exclusion dye were excluded and live cells (negative for the live/dead dye) were gated. Expanded CD45.2+ *Tet2/3* DKO T cells were sorted whereas CD45.1+ recipient cells were excluded. **Post-sort:** After sorting, a small quantity of the sample was assessed for purity.

Figure from Gioulbasani, Äijö et al, Communications Biology, 2024.

PMID: 39627458, Creative Commons CC-BY-NC-ND

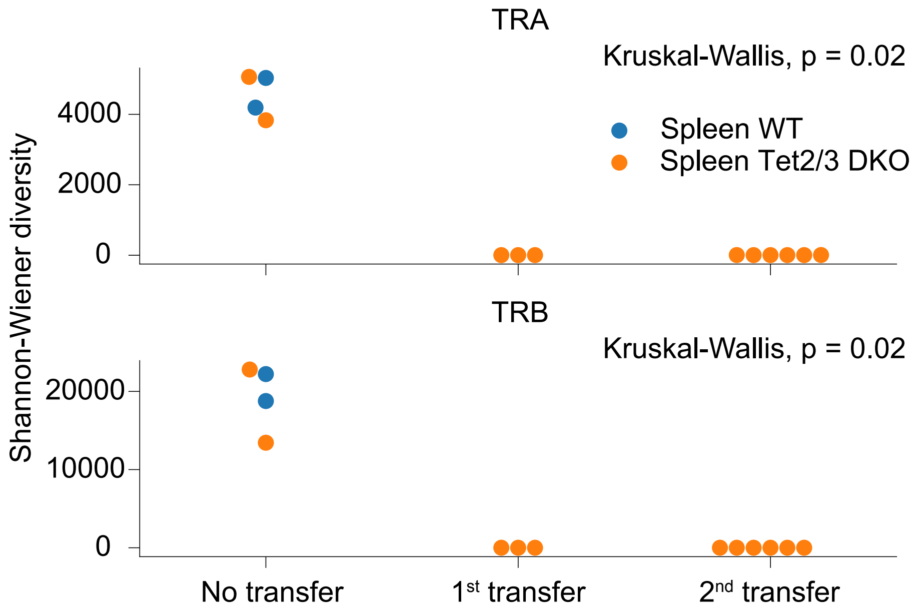

Supplementary Figure 11: **Reduced diversity of TCRα and TCRβ in *Tet2/3* DKO T cells after transfer compared to CD4 T cells in the spleen without transfer.** Shannon-Wiener diversity comparison focused on the clones assessed by TCR SMART-seq for CD4 cells isolated from the spleen of control CD4 cells, *Tet2/3* DKO CD4 cells or *Tet2/3* DKO CD4 cells that were transferred in congenic recipients (1st or 2nd transfer respectively). Statistical analysis using the Kruskal-Wallis test reveals that *Tet2/3* DKO T cells after transfer (1st, 2nd) exhibit significantly reduced diversity compared to

CD4 T cells (combined wild type and *Tet2/3* DKO CD4 T cells) isolated from spleen. P=0.02

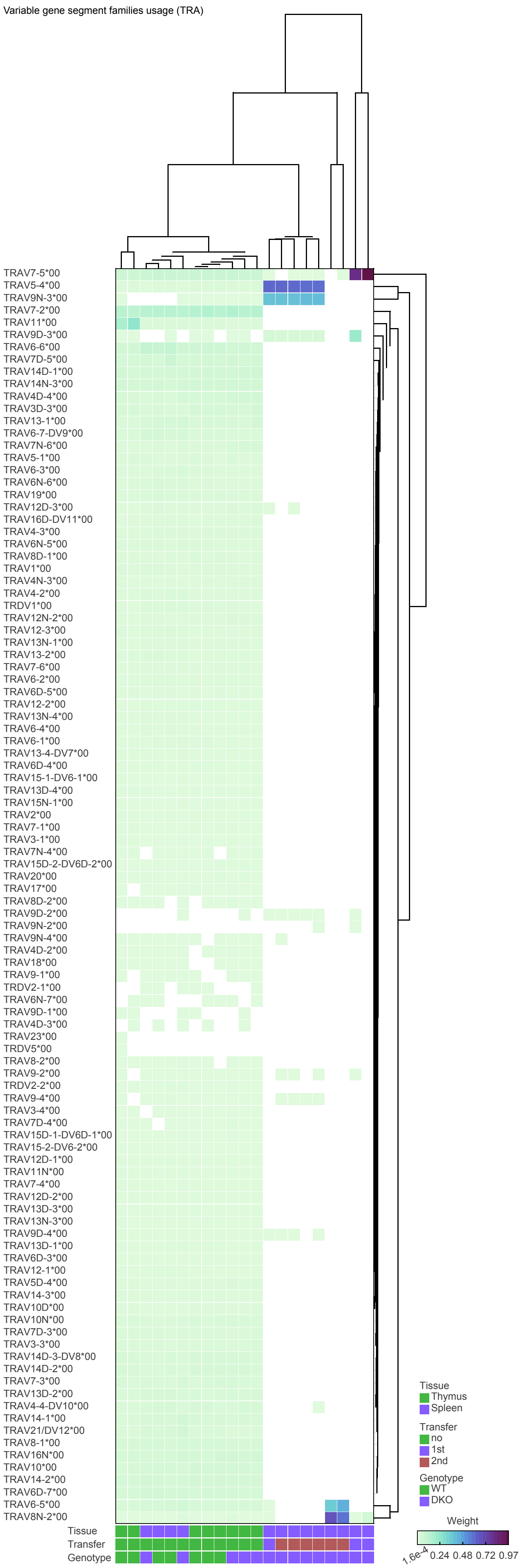

Supplementary Figure 12

Supplementary Figure 12: Comparison of diversity within V segment usage across TCRα samples reveals reduced diversity in *Tet2*/3 DKO T cells after transfer in congenic recipients.

Relative diversity was calculated between samples for the variable (V) segment gene usage for TCRα for WT CD4, *Tet2/3* DKO CD4 SP, WT CD4 isolated from spleen, *Tet2/3* DKO CD4 isolated from spleen, *Tet2/3* DKO CD4 after 1st or 2nd transfer in congenic recipients and plotted into heatmap. A hierarchical clustering was performed on the relative diversity weight. Parameters such as tissue (thymus/ spleen), transfer (no, 1st, 2nd) and genotype (WT, DKO) are indicated. Higher weight indicates greater contribution (as measured by read counts) in the overall V gene segment usage for the TCRα repertoire of the sample. The *Tet2/3* DKO samples for 1st and 2nd transfer have J gene segments with high contribution in the repertoire and thus reduced diversity.

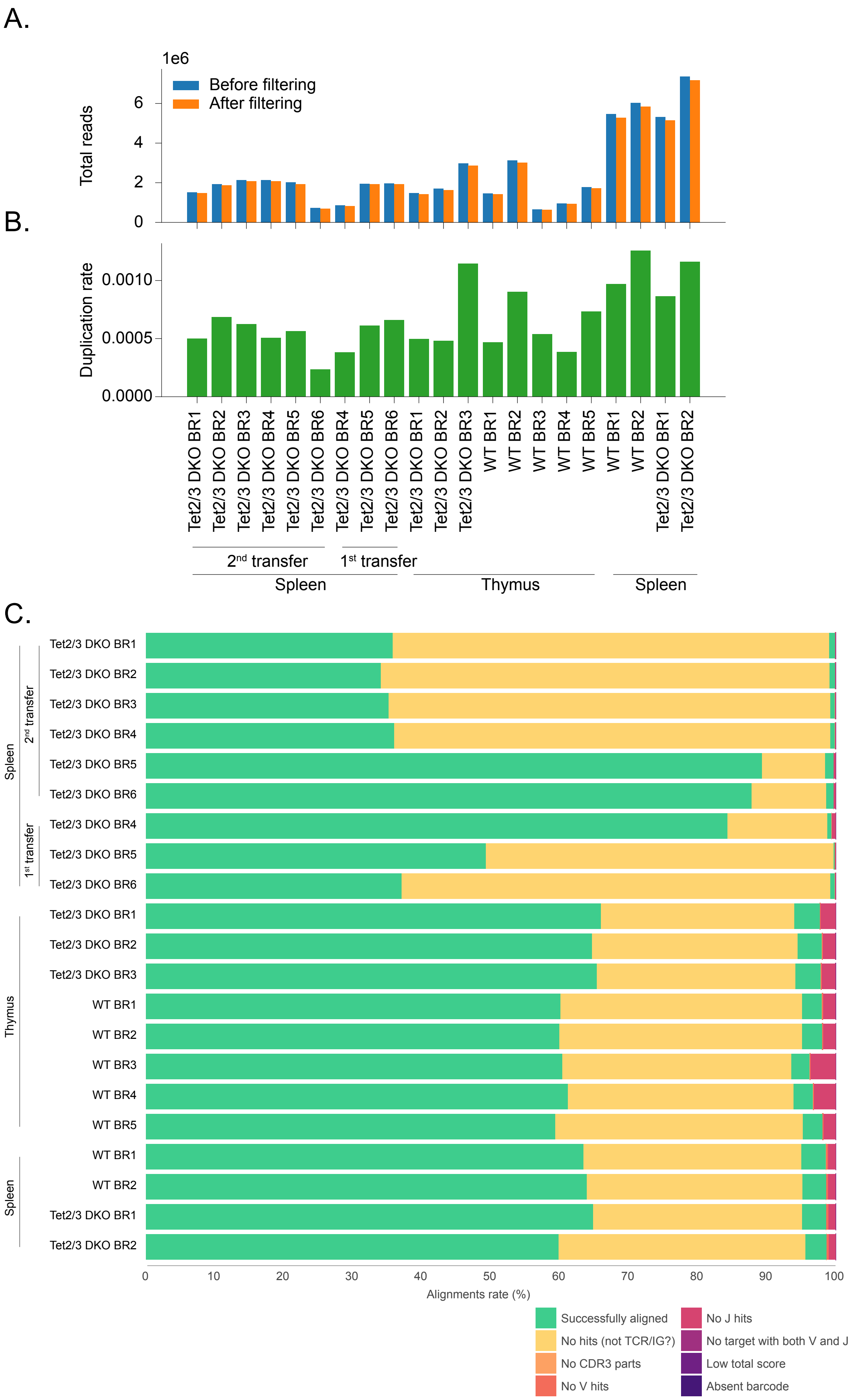

Supplementary Figure 13

Supplementary Figure 13: Sequencing metrics. A. Bar plot indicating the number of reads for each library before filtering (*in blue*) or after filtering (*in orange*). **B.** Bar plot indicating the number of duplicate reads (green bars) for each library. **C.** Bar plot indicating the alignment rate of reads for each library. Successfully aligned reads are indicated in green. Reads with no hits for the reference genome are shown in yellow. Reads that were not aligning to the J region are shown in pink. Reads that were not aligned to the hypervariable CDR3 region in light orange. Reads with no hits for the V region are indicated in bright orange.
